# The impact of odor–reward memory on chemotaxis in larval *Drosophila*

**DOI:** 10.1101/lm.037978.114

**Published:** 2015-05

**Authors:** Michael Schleyer, Samuel F. Reid, Evren Pamir, Timo Saumweber, Emmanouil Paisios, Alexander Davies, Bertram Gerber, Matthieu Louis

**Affiliations:** 1 Leibniz Institute for Neurobiology (LIN), Department Genetics of Learning and Memory, 39118 Magdeburg, Germany; 2 EMBL/CRG Systems Biology Research Unit, Centre for Genomic Regulation (CRG), 08003 Barcelona, Spain; 3 Universitat Pompeu Fabra (UPF), 08003 Barcelona, Spain; 4 University of Edinburgh, School of Informatics, Edinburgh EH8 9AB, United Kingdom; 5 Otto von Guericke University Magdeburg, Institute for Biology, Behavior Genetics, 39106 Magdeburg, Germany; 6 Center of Behavioural Brain Science (CBBS), Universitätsplatz 2, 39106 Magdeburg, Germany

## Abstract

How do animals adaptively integrate innate with learned behavioral tendencies? We tackle this question using chemotaxis as a paradigm. Chemotaxis in the *Drosophila* larva largely results from a sequence of runs and oriented turns. Thus, the larvae minimally need to determine (i) how fast to run, (ii) when to initiate a turn, and (iii) where to direct a turn. We first report how odor-source intensities modulate these decisions to bring about higher levels of chemotactic performance for higher odor-source intensities during innate chemotaxis. We then examine whether the same modulations are responsible for alterations of chemotactic performance by learned odor “valence” (understood throughout as level of attractiveness). We find that run speed (i) is neither modulated by the innate nor by the learned valence of an odor. Turn rate (ii), however, is modulated by both: the higher the innate or learned valence of the odor, the less often larvae turn whenever heading toward the odor source, and the more often they turn when heading away. Likewise, turning direction (iii) is modulated concordantly by innate and learned valence: turning is biased more strongly toward the odor source when either innate or learned valence is high. Using numerical simulations, we show that a modulation of both turn rate and of turning direction is sufficient to account for the empirically found differences in preference scores across experimental conditions. Our results suggest that innate and learned valence organize adaptive olfactory search behavior by their summed effects on turn rate and turning direction, but not on run speed. This work should aid studies into the neural mechanisms by which memory impacts specific aspects of behavior.

Larvae of the fruit fly *Drosophila melanogaster* possess a brain of only 10,000 neurons (Bossing et al. 1996; Larsen et al. 2009). Nonetheless, these animals display diverse capabilities of orientation including chemo-, photo-, and thermotaxis (Luo et al. 2010; Gomez-Marin et al. 2011; Gomez-Marin and Louis 2012, 2014; Kane et al. 2013; Klein et al. 2015) as well as associative learning and memory (for review, see Diegelmann et al. 2013; Schleyer et al. 2013). Here, we specifically investigate how an odor–reward memory influences innate chemotaxis.

The molecular and cellular bases of olfaction have been well characterized in the *Drosophila* larva, which possesses only 21 olfactory sensory neurons (Fishilevich et al. 2005; Kreher et al. 2005, 2008; Gerber and Stocker 2007), including detailed analyses of chemotaxis (Cobb 1999; Louis et al. 2008; Luo et al. 2010; Gomez-Marin et al. 2011; Lahiri et al. 2011; Gomez-Marin and Louis 2012, 2014; Gershow et al. 2012). Chemotaxis is characterized by alternating sequences of runs and oriented turns (Supplemental Fig. S1), and is largely modulated via three aspects of locomotion: how fast to run (run speed), when to initiate a turn (turn rate), and where to turn to (turning direction). Speed during runs is reported to be largely constant (Gomez-Marin et al. 2011; Gershow et al. 2012). Turn rate, however, is organized with respect to changes in odor concentration, such that instantaneous turn rate increases when odor concentration is decreasing during a run. Once a turn has been initiated, the larva scans the local odor gradient by casting its head from side to side. In the majority of the cases the larva then implements the next run into the direction of the odor source.

In addition to showing such innate chemotaxis, *Drosophila* larvae are able to associate odors with gustatory reinforcement, such as a sugar reward (Scherer et al. 2003; Neuser et al. 2005; Gerber and Hendel 2006). Upon pairing of an odor with sugar, larvae show enhanced preference toward that odor, while odor preference is decreased after unpaired presentations of the odor and reward (Saumweber et al. 2011a; Schleyer et al. 2011). However, exactly which aspects of locomotion are modulated by memory remains unknown.

In the present study, we compare the main control principles that bring about innate and learned chemotaxis. Specifically we examine whether the modulations of locomotion exerted by a learned odor are the same as those observed across different odor source intensities in innate behavior. Combined with a modeling perspective, this analysis sheds light on how associative memory is integrated with innate sensory-motor processing to organize adaptive orientation and search.

## Results

We studied olfactory orientation behavior of larval *Drosophila melanogaster* to understand how olfactory memories are integrated with innate chemotaxis. Specifically, we asked which modulations of behavior underlie the enhancement of innate odor preference observed for increasing odor source intensities, and whether modulations of the same sensory-motor features are responsible for the modulation of odor preference by associative olfactory memory.

### Preference behavior

**Figure 1.**
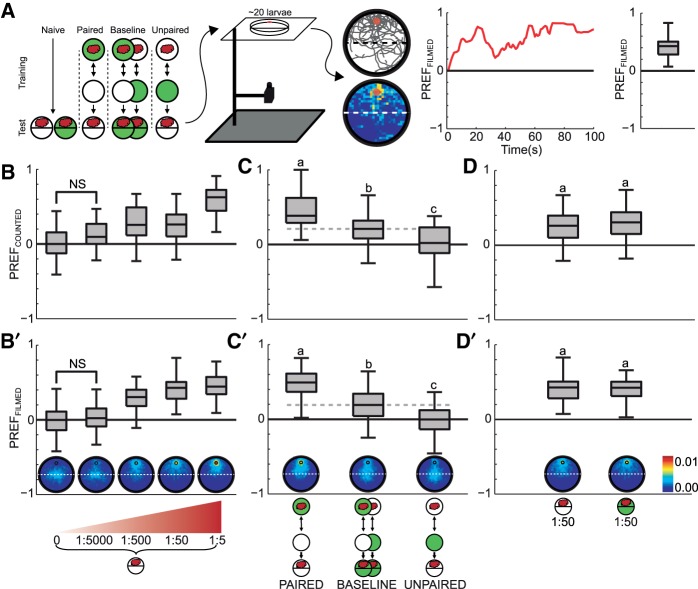
Olfactory preferences resulting from innate and learned behaviors. (*A*) Experimental design. Circles depict Petri dishes filled with an agarose substrate; their green fill denotes that a sugar reward (fructose) had been added to the substrate. The cloud illustrates the odor *n*-amyl acetate. The sample size for each experimental condition is 40, with each individual experiment being run with ∼20 *Drosophila* larvae. These groups received either no training (naïve, tested either in the absence or the presence of sugar), or received paired odor and sugar training or unpaired odor/sugar training before they were tested for olfactory preference. Associatively learned behavior is a form of learned search behavior, which is abolished in the presence of the sought-for sugar reward (e.g., Schleyer et al. 2011). Thus, testing in the presence of the sugar reward allows measuring baseline levels of olfactory behavior, without an impact of associative memories onto behavior (see body text for rational, as well as Supplemental Fig. S2). The second panel illustrates the experimental setup with the camera below the Petri dish; the light pad above the Petri dish is omitted for clarity. Within this setup, olfactory behavior was measured both by counting animals at the end of the test (i.e., after 5 min: PREF_COUNTED_) (*B*–*D*), and by video recording during the complete duration of testing (*B*′–*D*′). The third panel gives an example of the tracks (*top*) and density (*bottom*) of experimentally naïve larvae recorded during the 5-min testing period (dilution 1:50). The fourth panel shows how preference of the example sample evolves during the testing period; these data then are collapsed into one preference score (PREF_FILMED_) per sample. The fifth panel shows, for the 1:50 dilution also on display in (*B*′), the distribution of PREF_FILMED_ scores for 40 samples. (*B*,*B*′) Innate behavior. In experimentally naïve animals, olfactory preference is increased with increasing concentration of the odor source (*P* < 0.05, [*B*] *H* = 82.0, [*B*′] *H* = 112.2, df = 4, KW). Preference for the lowest odor concentration does not differ from the no-odor control (*P* > 0.05/4, [*B*] *U* = 551.5, [*B*′] *U* = 701.5, MWU) (this is indicated by NS); preferences are significantly different from control for all higher concentrations (*P* < 0.05/4, [*B*] *U* = 320, 381.5, 75, [*B*′] *U* = 206, 101, 37, MWU). (*C*,*C*′) Learned behavior. Olfactory preference is affected by training experience (*P* < 0.05, [*C*] *H* = 44.4, [*C*′] *H* = 64.4, df = 2, KW). When tested on pure agarose, larvae show a higher preference after paired than after unpaired training (*P* < 0.05/3, [*C*] *U* = 181, [*C*′] *U* = 104, MWU). Animals tested in the presence of fructose display intermediate baseline preference (*P* < 0.05/3, [*C*] *U* = 698, 413, [*C*′] *U* = 1014.5, 936, MWU). (*D*,*D*′) Olfactory preference in experimentally naïve animals is not affected by the presence of fructose (*P* > 0.05, [*D*] *U* = 673, [*D*′] *U* = 782, MWU). Bold lines show medians, the box boundaries the 25% (*q*_1_) and 75% (*q*_3_) quartiles, and the upper whisker: *q*_3_ + 1.5 × (*q*_3_ − *q*_1_) and lower whisker: *q*_1_ − 1.5 × (*q*_3_ − *q*_1_). Significant between-group differences (Mann–Whitney *U*-tests: MWU) are indicated with different lower case letters above the boxes in *C*,*C*′. Sample size (*N*), featuring approximately *n* = 20 larvae per sample, is *N* = 80 for the baseline condition and *N* = 40 for all other conditions.

We first determined innate odor preference as a function of the concentration of the odor source. We did so either by end-point counting, that is by counting the numbers of larvae after a choice period of 5 min (PREF_COUNTED_), or by determining the preference via the proportion of time spent on either half of the dish throughout the entire 5-min testing period (PREF_FILMED_) (Fig. 1A). Both types of score revealed that innate preference increased with increasing odor-source concentration (Fig. 1B,B′). Next, we examined how an associative olfactory memory modulates odor preference.

To address this question it was important that larvae are capable of behaviorally expressing an associative memory—or not. That is, after paired odor–reward training odor preference was higher than after unpaired presentations of odor and reward (Fig. 1C,C′ and note in Materials and Methods), a behavior revealing associative memories. This difference in preference was abolished in the presence of the sugar reward (Supplemental Fig. S2; Gerber and Hendel 2006; Saumweber et al. 2011a; Schleyer et al. 2011, 2015). Learned behavior thus can be grasped as learned search that ceases in the presence of the sought-for sugar reward. Notably, innate olfactory behavior remains unaltered in the presence of the sugar reward (see next paragraph). This offers the opportunity to measure baseline levels of olfactory behavior without the behavioral influence of associative memories, simply by running the test in the presence of the sugar reward. We found that relative to this baseline odor preference was increased after paired training of odor and reward and was decreased after unpaired training (Fig. 1C,C′). Within the present data set, preference scores were reduced to zero after unpaired training (rightmost box plots in Fig. 1C,C′); it will become important in the Discussion that when experiments are performed at overall lower levels of baseline preference, unpaired training can result in repulsion to the odor for the unpaired group (e.g., Saumweber et al. 2011a, Fig. 6; Schleyer et al. 2011, Supplemental Fig. S2).

Given that the measurement of baseline preference involved testing the trained animals in the presence of the sugar reward, we asked whether the presence of the reward also had an influence on innate odor preference. We found this not to be the case (Fig. 1D,D′; Hendel et al. 2005; Schleyer et al. 2011, 2015). In conclusion, learned but not innate olfactory preference is affected by the presence of the reward.

We next asked by which particular behavioral processes learned preference comes about, and compare them to those behavioral processes underlying innate preference. Given that *Drosophila* larvae orient in an odor gradient through a sequence of runs and turns, we considered the modulation of three general aspects of the orientation behavior: (i) how fast to run (run speed), (ii) when to initiate a turn (turn rate), and (iii) where to turn to (turning direction).

### Run speed

**Figure 2.**
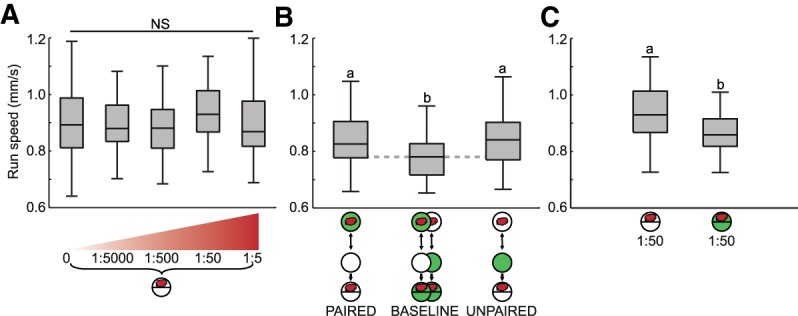
Run speed. (*A*) Innate behavior. In experimentally naïve animals, run speed is not influenced by the concentration of the odor source (*P* > 0.05, *H* = 5.6, df = 4, KW) (indicated by NS). (*B*) Learned behavior. After training, we find differences in run speed between experimental groups (*P* < 0.05, *H* = 18, df = 2, KW). These differences are nonassociative in nature, as both reciprocally trained groups tested on pure agarose display higher run speed than baseline (*P* < 0.05/3, *U* = 981, 976, MWU), but do not differ from each other (*P* > 0.05/3, *U* = 794, MWU). (*C*) In experimentally naïve animals, run speed is decreased by the presence of fructose (*P* < 0.05, *U* = 513, MWU). For other details, see legend of Figure 1.

Regarding innate olfactory behavior, we found no systematic increase in run speed when using odor sources of increasing concentration (Fig. 2A; Supplemental Fig. S3). Likewise, run speed was equal after paired and unpaired training (leftmost versus rightmost box plot in Fig. 2B). Thus neither the innate nor the learned valence of an odor modulates run speed during chemotaxis (“valence” defined throughout as the degree of attractiveness).

In contrast, run speed was decreased in the presence of the sugar reward, both in experimentally naïve larvae (Fig. 2C) and in trained larvae (middle box plot in Fig. 2B). Thus, gustatory behavior, at least in part, operates on changes of run speed and it may be viewed as a form of *kinesis* (Fraenkel and Gunn 1961): when the gustatory situation is “good,” slowing down its runs helps the larva to not drift away from a food source. In contrast, behavior toward an attractive odor involves a modulation of the direction of motion according to a form of *taxis* (Fraenkel and Gunn 1961; see next two sections). We note that across the trained groups (paired, baseline, and unpaired conditions) run speed was generally lower than in experimentally naïve larvae (compare Fig. 2A versus B), possibly due to effects of handling stress, stimulus exposure, fatigue, or a combination of these factors.

### Turn rate

**Figure 3.**
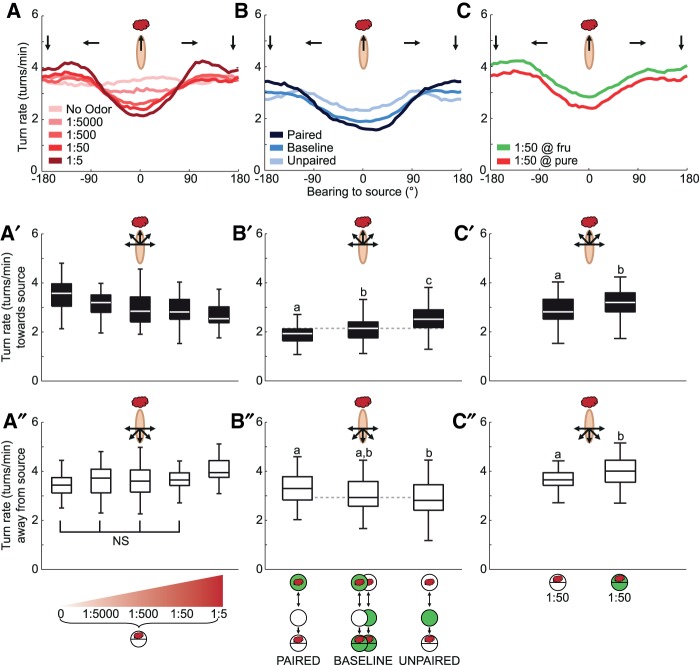
Turn rate. Turn rate as function of bearing angle to the odor source (*A*–*C*), and summarized by bearing angles toward (*A*′–*C*′) or away from (*A*″–*C*″) the odor source. (*A*–*A*″) Innate behavior. In experimentally naïve animals, increasing the concentration of the odor source decreased turn rate when heading toward the odor source (*A*′) (*P* < 0.05, *H* = 41.8, df = 4, KW), and increased turn rate when heading away from it (*A*″) (*P* < 0.05, *H* = 23.4, df = 4, KW). When heading toward the odor source (*A*′), turn rate for all odor concentrations differ from the no-odor condition (*P* < 0.05/4, *U* = 516, 351, 357, 225, MWU). When heading away from the source (*A*″), turn rates differ from control only for the highest concentration (*P* < 0.05/4, *U* = 315, MWU), but not for lower concentrations (*P* > 0.05/4, *U* = 637, 674, 612, MWU). (*B*–*B*″) Learned behavior. As compared with baseline, paired and unpaired training modulate turn rate in opposing ways; these effects, as in the case of innate behavior, differ in sign across bearing angles: when heading toward the odor source (*B*′) (*P* < 0.05, *H* = 26.3, df = 2, KW), turn rates after paired training are lower than after unpaired training (*P* < 0.05/3, *U* = 304, MWU) and lower than baseline (*P* < 0.05/3: *U* = 1152, MWU); after unpaired training turn rates are higher than baseline (*P* < 0.05/3: *U* = 938, MWU). When heading away from the odor source (*B*″), the results are inverse (*P* < 0.05, *H* = 6.9, df = 2, KW), that is turn rates after paired training are higher than after unpaired training (*P* < 0.05/3, *U* = 544, MWU); relative to baseline, turn rates tend to be higher after paired and lower after unpaired training (*P* > 0.05/3: *U* = 1224, 1449, MWU). (*C*–*C*″) In experimentally naïve animals, turn rate is generally increased in the presence of the reward, regardless of bearing angle (*P* < 0.05, *U* = 547, 529, MWU). For other details, see legend of Figure 1.

One behavioral process by which innate chemotaxis comes about is that larvae turn less frequently when heading toward the odor source (absolute bearing angle <90°), and more frequently when heading away from it (absolute bearing angle >90°) (Gomez-Marin et al. 2011; Gershow et al. 2012). Both these effects became more pronounced for higher odor source concentrations (Fig. 3A–A″; Supplemental Fig. S4). We therefore asked whether the larvae show corresponding modulations of turn rate for learned odors.

When heading toward the odor source, the larvae decreased turn rate after paired training, and increased turn rate after unpaired training (Fig. 3B,B′; Supplemental Fig. S4). Conversely, when heading away from the odor source, larvae increased turn rate after paired training and showed a tendency to decrease turn rate after unpaired training (Fig. 3B,B″; Supplemental Fig. S4). Thus, both when heading toward and when heading away from the odor source, memories after paired and after unpaired training modulate the decision to initiate a turn, in respectively opposite ways. We note that for both innate and learned chemotaxis, the total turn rate was unchanged (Supplemental Fig. S5); however, in the presence of sugar the total turn rate was increased, regardless of where the larvae were heading to (Fig. 3C–C″; Supplemental Figs. S4, S5) (for turn rate data separated by bearing angle and distance to the odor source, see Supplemental Fig. S6).

To summarize, both the innate and the learned valence of an odor influenced turn initiation in a similar way: in both cases high levels of chemotaxis came about by decreases in instantaneous turn rate when heading toward the odor source and by increases in turn rate when heading away from the source. Conversely, low levels of chemotaxis came about by increases in turn rate when heading toward and decreases when heading away from the odor source.

### Turning direction

**Figure 4.**
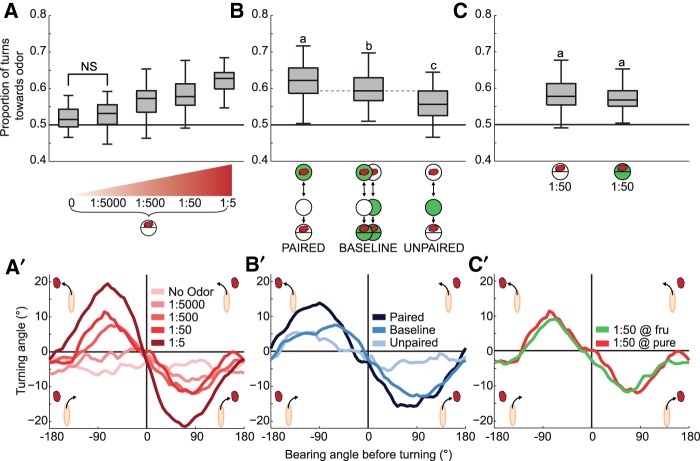
Turning direction. (*A*,*A*′) Innate behavior. As odor source concentration is increased, experimentally naïve animals allocate the more of their turns toward, rather than away from, the odor source (*P* < 0.05, *H* = 98.9, df = 4, KW). Turning toward the lowest odor concentration does not differ from the no-odor control (*P* > 0.05/4, *U* = 656, MWU), but is significantly different from control for all higher concentrations (*P* < 0.05/4, *U* = 311, 198, 24, MWU). In (*A*′) average turning angles are plotted across bearing angle before the turn. At the *upper left*, for example, one can see that animals turn more to the left if at the moment of turn initiation the local odor gradient points toward their left side; the same is the case for turns toward the right. These modulations are the more pronounced the higher the concentration of the odor source. Whenever heading directly toward or away from the odor source (bearing angles of 0° or 180°), the animals are equally likely to turn left and right, resulting in average turning angles of 0°. (*B*,*B*′) Learned behavior. Associative training influences the proportion of turns toward odor (*P* < 0.05, *H* = 28.3, df = 2, KW). Specifically, after paired training the animals implement more of their turns toward the odor source than after unpaired training (*P* < 0.05/3, *U* = 286, MWU). These memory-based modulations are significant also relative to baseline (*P* < 0.05/3: *U* = 1142.5, 905.5, MWU), and can be discerned when plotting average turning angles across the bearing angle before the turn (*B*′). (*C*,*C*′) In experimentally naïve animals, the proportion of turns toward the odor source is not affected by the presence of the reward (*P* > 0.05, *U* = 672.5, MWU). For other details, see legend of Figure 1.

Innate chemotaxis results in part from the ability of the larvae to turn more frequently toward than away from the odor (Gomez-Marin et al. 2011; Gershow et al. 2012). Accordingly, for higher odor source concentrations we observed a higher fraction of turns toward the source (Fig. 4A,A′). After learning, the decision where to turn to was affected in a similar way: compared with baseline the proportion of turns toward the odor source was increased after paired training, while larvae that had received unpaired training showed a decrease in the proportion of turns toward the odor source (Fig. 4B,B′). We note that the strongest between-group effects were seen when larvae are orientated orthogonal to the odor source, in other words for bearing angles around −90° and +90° (Fig. 4; Supplemental Fig. S7; Gomez-Marin et al. 2011; Gershow et al. 2012). During innate chemotaxis, no modulation of turning direction was found in the presence of sugar (Fig. 4C,C′; Supplemental Fig. S7).

To summarize, associative odor memories influenced turning direction in the same way as innate chemotaxis: the higher the valence of the odor, either due to an increase in odor source concentration during innate chemotaxis, or based on paired odor–sugar training, the stronger the bias in the direction of turning toward the odor source. The opposite trend was observed when valence was lowered either by using lower odor source concentrations or after unpaired training.

### Modeling

Our analyses uncovered a significant modulatory effect of memory on two key features of larval chemotaxis, namely of turn rate and of turning direction. We next wondered whether these modulations would be sufficient to bring about the observed “macroscopic” between-group differences in odor preference scores (i.e., PREF_FILMED_). We devised a deliberately minimalistic model of larval chemotaxis in which run speed, turn rate and turning direction were parametrically estimated from the experimental data (see Materials and Methods). Specifically, we assumed that turn rate and turning direction are functions of two variables, the current bearing angle toward the odor source and the current distance from it (displayed in Supplemental Figs. S6, S7), as these appear to be the main determinants of sensory input during chemotaxis. Note that we estimated the parametric dependency of turn rate and turning direction separately for each of the 10 experimental groups, and that we used a constant run speed for each experimental group (i.e., the respective groups’ median values of run speed as shown in Fig. 2).

**Figure 5.**
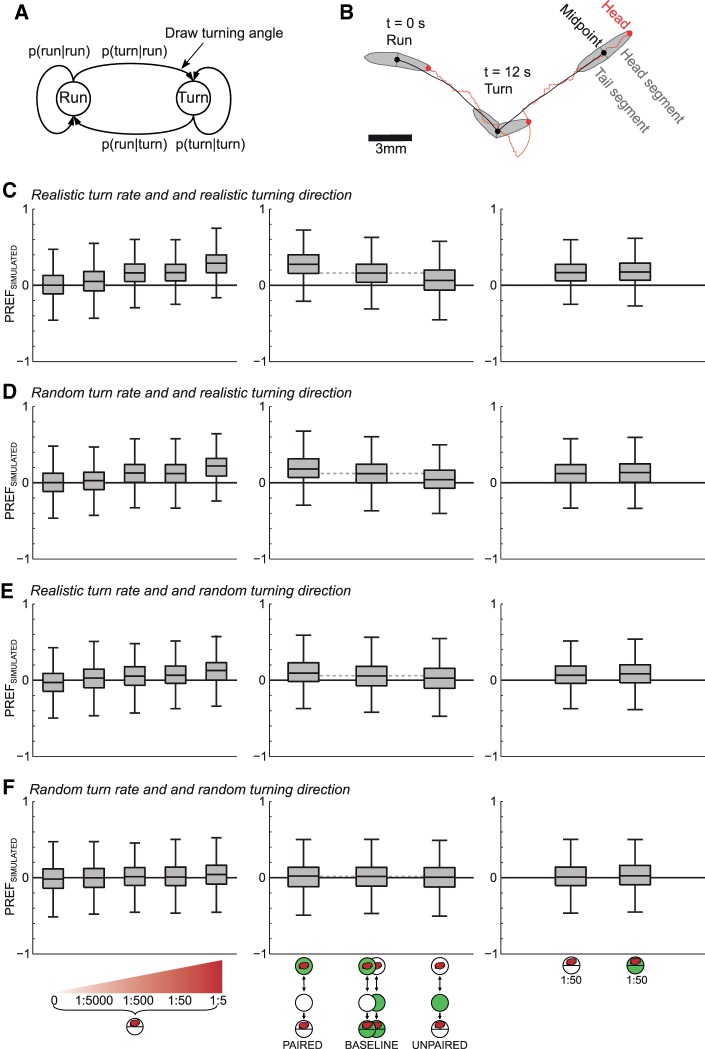
Model. (*A*) Larval behavior is simulated as a two-state Markov process. While in the run state, animals move forward with constant run speed. At each simulation time step, model animals may switch to the turn state with probability *P*(turn|run) = *r*_turn_ · d*t*, where *r*_turn_ is the turn rate and d*t* = 0.0625 sec is the simulation time step. For each turn a turning angle is drawn from a subsample of the empirical turning angles (see Materials and Methods for details). While in the turn state model larva cast the head segment in the direction of the turning angle. Turning is terminated deterministically with probability *P*(run|turn) = 1 when the angle between the tail and the head segment equals the turning angle. In the simulations, run speed was set to the median empirical run speed of the respective experimental group. (*B*) Contours of simulated larvae sketched at three different time points along a model trajectory of 24 sec. Simulated larvae consist of a head and a tail segment. Upon turning (*t* = 12 sec) the head segment swings laterally until the designated turning angle is reached. (*C*–*F*) Simulated preference indices of four different model modes. For each mode and condition, 1000 trials have been simulated where one trial consists of 13 animals simulated over 5 min. The model mode with realistic turn rate and realistic turning direction (*C*) best reproduces the observed differences between the experimental groups (cf. Fig. 5C and Fig. 1*B*′–*D*′). (*D*,*E*) Show the models’ performance when either turn rate (*D*) or the angle of turning direction (*E*) was randomized. In (*F*) both the turn rate and the angle of turning direction was randomized.

Our model was run in four different modes: in the first mode (*realistic turn rate and realistic turning direction*), both the turn rate and the angle of turning direction were estimated from the empirical data of the respective experimental condition. We found that such a model was largely sufficient to reproduce the pattern of odor preference across experimental groups (compare Fig. 1B′–D′ with Fig. 5C). The model accounted for the increase in innate odor preference observed for increasing concentrations of the odor source (Fig. 5C, left panel). More important, it led to a symmetric offset in preference between reciprocally trained groups relative to baseline (Fig. 5C, middle panel), as well as no difference in preference between animals tested on pure agarose versus on fructose (Fig. 5C, right panel). Thus, the combined modulation of turn rate and of turning direction is sufficient to account for the empirically found differences in preference across experimental conditions. This made us wonder whether indeed both types of sensory-motor modulation are required.

We therefore probed the contribution of modulating the turn rate and turning direction independently. In the second mode of the model (*random turn rate and realistic turning direction*), the empirical turn rate was substituted by random turn rates, while in the third mode (*realistic turn rate and random turning direction*), the empirical angle of turning direction was substituted by random angles of turning. We found that none of these simplified model modes could fully reproduce the across-group differences in preference scores obtained with the full model (cf. Fig. 5C versus D,E). However, randomizing the empirical distribution of turning angles had an apparently stronger negative effect on the fit with the experimental results than randomizing the turn rate (Fig. 5D versus E). As expected, when both turn rate and turning direction were randomized, the model led to random spatial orientation (Fig. 5F).

In summary, the fit between numerical simulations and experimental observations indicates that the modulation of when-to-turn and where-to-turn-to decisions are major mechanisms for the modulation of innate and learned chemotaxis. This does not exclude that changes in other aspects of locomotion also contribute to modulations of chemotaxis, but likely these modulations are not strictly necessary.

## Discussion

### A “baseline” against which to measure associative memory

A fundamental issue for any study of memory is that a baseline is required against which memory effects can be assessed. The paradigm used in the present study offers a solution by measuring olfactory preference of larvae that have an associative memory, but do not behaviorally express it (Gerber and Hendel 2006; Saumweber et al. 2011a; Schleyer et al. 2011, 2015). That is, learned behavior in the present paradigm is a search for reward. After paired training, the larvae search for the reward where the odor *is*, while after unpaired training they search for the reward where the odor *is not*. In line with theoretical considerations (e.g., Craig 1918; Elsner and Hommel 2001; Hoffmann 2003), such search for reward is suppressed if the sought-for reward is present (Supplemental Fig. S2). In contrast, innate olfactory preference, to the extent tested, is unaffected by the presence of the reward (Fig. 1D,D′; Schleyer et al. 2011, 2015). Thus, larvae trained in either a paired or unpaired manner and tested in the presence of the reward provide a baseline against which the behavioral impact of associative olfactory memory can be assessed.

Obviously, the presence of the reward is not without behavioral effect: run speed is decreased (Fig. 2C; see also Fig. 2B), and total turn rate is increased (Supplemental Fig. S5C). However, neither a general decrease in run speed nor a general increase in turn rate can as such orient the animal toward the odor source. Thus, the presence of the reward does not impact the innate olfactory preference (Fig. 1D,D′; see also Fig. 5).

### Innate and learned valence modulate the same aspects of locomotion, in the same way

This study was undertaken to examine how associative odor memories are integrated with innate chemotaxis to organize adaptive search behavior. We concentrated on three behavioral features (Gomez-Marin et al. 2011; Gershow et al. 2012), each with the potential to direct larvae toward or away from the odor source:

- “Run speed.” A larva may speed up when heading toward an odor (i.e., when odor concentration increases along its path), and slow down when heading away.
- “Turn rate.” A larva may turn less often when heading toward the odor source (i.e., when odor concentration increases along its path), and more often when heading away from it.
- “Turning direction.” A larva may collect information about the direction of the odor source during its run and/or during the large-amplitude head casts flanking a turn, and use this information to turn more often into the desired direction.

Regarding innate behavior, we confirmed that larval *Drosophila* modulate turn rate and turning direction, but not run speed (Gomez-Marin et al. 2011). We extend these findings by showing that modulations of both turn rate and turning direction, but not of run speed, underlie the adjustment of innate odor preference across four orders of magnitude in odor source concentration (Figs. 2A, 3A′,A″, 4A, 5C).

Regarding learned behavior, we find that the turn rate and turning direction are modulated in the same way: after paired odor–sugar training odor preferences are increased (because the odor predicts where sugar *is*), while after unpaired presentations of the odor and the sugar reward preferences are decreased (because the odor predicts where the sugar *is not*). These opposite, contingency-dependent modulations of preference are brought about by, respectively, opposite modulations of both turn rate and turning direction, but not of run speed (Figs. 2B, 3B′,B″, 4B, 5C). Notably, relative to baseline, the instantaneous turn rate while heading toward the odor is decreased after paired and increased after unpaired training (Fig. 3B′), while when heading away from the odor the opposite effects are observed (Fig. 3B″). Likewise, the proportion of turns toward the odor source is increased after paired and decreased after unpaired training (Fig. 4B). Thus, both paired and unpaired training do induce memory, and these respective memories impact the same behavioral features in opposite ways.

Our modeling approach (Fig. 5) provides a sanity check for these conclusions by showing that realistic modulations of turn rate and of turning direction are sufficient to reproduce the empirical differences in innate preference for odor sources of different concentration, as well as for the differences in preference between paired and unpaired trained groups (Fig. 1B–D′). Thus, the alternation between runs and turns is modulated in the same way by the innate and the associatively learned valence of an odor. In this specific respect, innate and learned valence is of the same nature (see also Brembs and Heisenberg 2000).

### A behavior systems-level perspective

**Figure 6.**
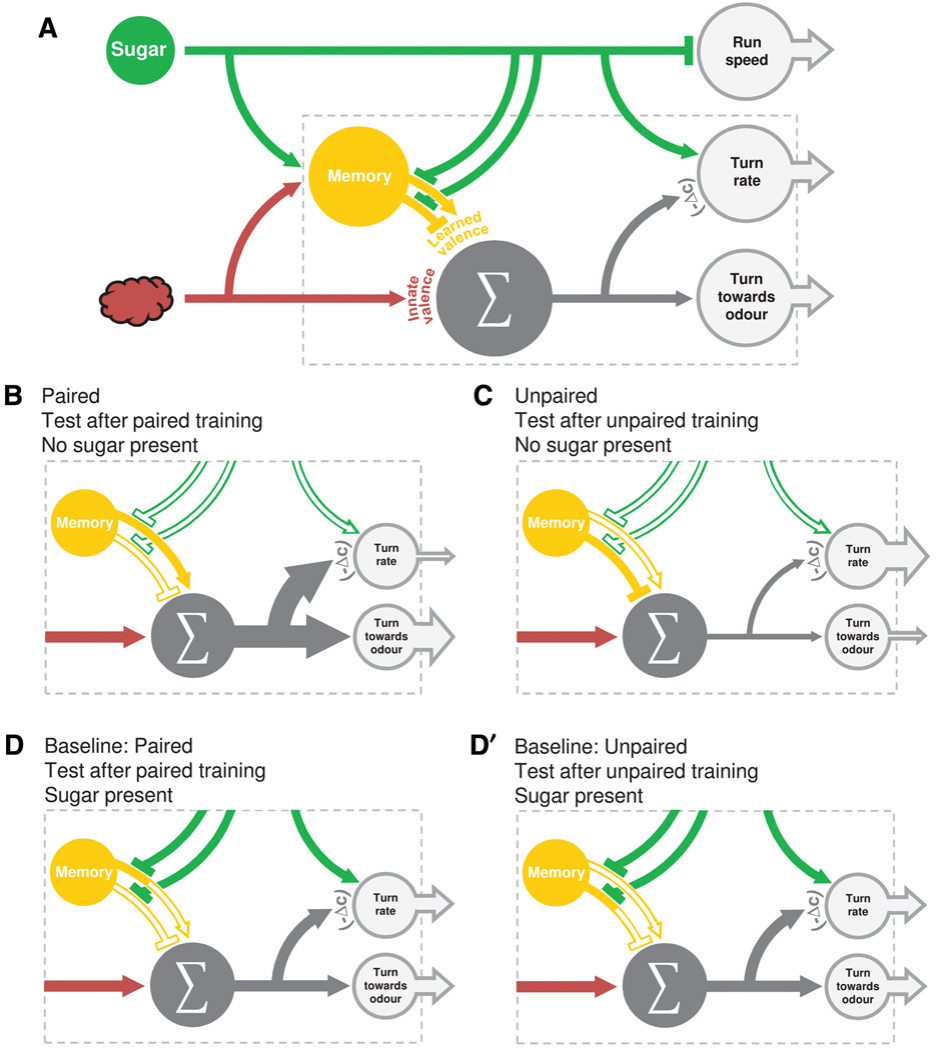
Working hypothesis. Working hypothesis of how sugar, odor and odor–sugar memory modulate chemotaxis. (*A*) An overview, (*B*–*D*) illustrates the plausible outcome of different training procedures. (*A*) Sugar reduces run speed and increases turn rate. Odor signals are processed toward the motor system via two routes. First, most odors elicit a response with a positive valence in experimentally naïve larvae (valence understood throughout as level of attractiveness). Second, during associative training a memory is formed. This memory is of positive valence after paired odor–sugar training, and of negative valence after unpaired presentations of odor and sugar. At the moment of testing, learned and innate valences are summed, and the resulting signal modulates turn rate and turning direction. We propose that a multiplication rule involving the negative change in odor concentration, that is −(▵*c*), ensures that a net positive valence reduces turn rate when approaching the odor source, and increases turn rate when moving away from it. Notably, signaling of learned but not of innate valence can be blocked by the presence of sugar in the test situation. (*B*) After paired training, a positive learned valence is added to the innate valence of the odor. The summed valence leads to an increase in the proportion of turns toward the odor compared with baseline (*D*). Furthermore, when the larva is approaching the odor source (▵*c* is positive), turn rate decreases. (*C*) After unpaired training, negative learned valence is added to innate valence. If the innate valence remains larger than the negative learned valence, the combined outcome still modulates turn rate and turning direction positively, even though the degree of attraction is reduced compared with paired training (*B*). If the level of innate valence were lower than in the present experimental condition (and thus positive innate valence would be smaller than negative learned valence), we would expect the sum of innate and learned valence to be negative after unpaired training. This would lead to aversion to the odor. (*D*,*D*′) When tested in the presence of sugar, signaling of learned valence is blocked. Thus, only innate valence determines chemotaxis and therefore larval behavior is the same after paired and unpaired training. In addition, the presence of sugar increases the overall turn rates in both these groups.

Based on the above analyses, the impact of associative memory clearly does not leave a distinct “footprint” on the level of the sensory-motor strategies. Instead, learned valence and innate valence apparently summate and feed into a common descending pathway organizing behavior toward odors (Fig. 6). Are, thus, learned and innate behavior “just the same?” On what one may call a systems-level organization of behavior, this is clearly not the case: depending on the circumstances of testing, learned valence can influence behavior—or not. That is, learned modulations of behavior are abolished by the presence of the reward during the test (Supplemental Fig. S2; Gerber and Hendel 2006; Saumweber et al. 2011a; Schleyer et al. 2011, 2015). This ensures that memory results in an active search organized toward its outcome, namely finding the food reward. Such an active search strategy adaptively ceases whenever the sought-for object has already been found. In contrast, innate valence is rather responsive and is expressed largely independent of the circumstances of testing.

### Do associative memories feed back onto sensory processing?

The observation that preference scores are reduced to zero in the unpaired group (Fig. 1C,C′) may imply that the negative-valence memory established by unpaired training blocks odor processing altogether (Twick et al. 2014). Such a block, however, could not account for the repulsion observed after unpaired training under conditions of overall lower preference (e.g., Supplemental Fig. S8; Chen et al. 2011; Saumweber et al. 2011a; Schleyer et al. 2011; Mishra et al. 2013). In contrast, the summation scenario we propose can account for such odor repulsion after unpaired training, if the level of innate valence were smaller than the negative learned valence. It can also seamlessly be extended to account for the repulsion after paired odor-punishment training (e.g., Schleyer et al. 2011). We therefore favor a scenario in which learned and innate valence summate to govern larval chemotaxis by specifically modulating turn rate and turning direction. The present analysis offers a conceptual framework for upcoming analyses of these processes at the neuronal level, and may facilitate the design of biologically inspired technical devices for autonomous search.

## Materials and Methods

### General

Canton-S wild-type *Drosophila melanogaster* larvae were used for all experiments. Larvae were maintained on conventional cornmeal-agar molasses medium at 22°C, 60%–70% relative humidity, in a 12 h light/dark cycle. Experiments were performed on third instar foraging larvae, at a room temperature of 20°C–24°C. For all experiments, larvae were removed from the food medium and washed briefly in distilled water before the start of experiments. The training and testing of larvae was carried out in 15-cm diameter Petri dishes (Sarstedt), which were prefilled with 1% agarose (SeaKem LE Agarose, Lonza) and stored at 4°C until used. To create a sweet, rewarding substrate, 0.2 M fructose (FRU; CAS No 57-48-7; 99% purity; Sigma-Aldrich) was added to the agarose. The odor (*n*-amyl acetate; AM; CAS No 628-63-7; 99% purity; Sigma-Aldrich) was presented by placing a 10 µL droplet within a transparent reinforcement ring that was fixed onto the inner side of the Petri dish lid (Supplemental Fig. S1A). Dilutions were made in paraffin oil (CAS No 8012-95-1; Sigma-Aldrich) as indicated in the Results. For all experiments, odor gradients were established for 1 min prior to the introduction of the larvae.

### Innate olfactory behavior

A group of ∼20 larvae was placed in the 4.50 × 0.85 cm starting zone of the Petri dish (Supplemental Fig. S1A). To create a choice situation, the Petri dish contained only one odor source, which was placed 4.5 cm from the midline of the dish (Supplemental Fig. S1A). The dish was closed with a lid and placed under a light pad (Slimlite, Kaiser Fototechnik). Larval behavior was filmed for 5 min from below for offline analyses (camera: Scout SCA1390-17FC, Basler) (Supplemental Movies S1–3). After 5 min the dish was removed and the position of larvae was scored as being either on the odor side of the dish, the no-odor side, or in a 1-cm-wide neutral zone (Supplemental Fig. S1A).

### Conditioned olfactory behavior

Larvae underwent one of two possible training protocols (Fig. 1A): either *n*-amyl acetate (AM; red cloud in Fig. 1A) was presented with the rewarding fructose substrate (+; green fill of Petri dish in Fig. 1A), followed by a blank trial featuring exposure to a Petri dish without fructose and without odor (AM+/blank). This is henceforth called paired training and is abbreviated as AM+. Alternatively, the larvae were trained reciprocally such that AM and the reward were presented on separate trials. This is henceforth called unpaired training and is abbreviated as AM/+. For the test, larval behavior toward AM was recorded.

As an example, consider the paired training protocol (AM+). Both reinforcement rings on the lid were loaded with AM diluted 1:50 in paraffin oil to ensure that odor was present throughout the entire Petri dish. For the first training trial, larvae were placed into the starting zone of a fructose-containing Petri dish and covered with the lid that contained the two sources loaded with AM. After 5 min they were transferred to a fresh dish in which no odor was presented and no fructose had been added to the substrate. This training cycle was repeated two more times. Animals were then placed in the starting zone of a Petri dish with AM loaded on only one side of the Petri dish in order to create a choice situation. This test plate did not contain fructose, unless otherwise stated. Larval behavior data were then acquired as described above. The second group of animals was trained reciprocally (AM/+), i.e., odor and reward were presented in an unpaired way, and larvae were tested as mentioned.

In half of the cases the sequence of training trials was as indicated (i.e., AM+/blank and AM/+), and in the other half of the cases the sequence of training trials was reversed (i.e., blank/AM+ and +/AM). Note that the sequence of training trials does not have an effect on behavior at the time of the test (Schleyer 2009; Saumweber et al. 2011b).

### “Baseline” behavior after training

To characterize the impact of memory on olfactory behavior, a behavioral baseline is needed with respect to which memory effects can be assessed. Learned behavior after odor–reward training constitutes a search for reward that is suppressed if the sought-for reward is present during the test (Gerber and Hendel 2006; Saumweber et al. 2011a; Schleyer et al. 2011) (innate olfactory preference is not affected by the presence of the sugar reward: Fig. 1D,D′; Schleyer et al. 2011). We thus trained larvae in either a paired or unpaired manner, and tested them for their odor preference in the presence of the reward. In Supplemental Figure S2 we present their preference scores, and all other measures of their olfactory behavior, separated by training. These results justified the pooling of data from these groups to estimate baseline olfactory behavior with no measurable influence of associative olfactory memory.

We emphasize that experimentally naïve animals cannot be used to provide a reliable baseline to measure associative memories. This is because the nonassociative influences of animal handling, of odor exposure, and of sugar exposure in the trained but not the naïve larvae can confound such a comparison (Rescorla 1967; Quinn et al. 1974; Lieberman 2004) (in particular odor exposure effects are well documented for larval *Drosophila*: Cobb and Domain 2000; Boyle and Cobb 2005; Colomb et al. 2007; Larkin et al. 2010). Given that paired, unpaired and baseline groups are all equated for these aspects of exposure, such exposure effects are not immediately plausible explanations for behavioral differences between these experimental conditions.

### A note on the use of the terms “reward” and “punishment”

The terms “reward” and “punishment,” strictly speaking, are reserved for operant, rather than classical, conditioning processes (e.g., Brembs and Heisenberg 2000). Within the present paper, which uses a conditioning paradigm that is likely largely classical of nature, we adopt these terms in a liberal way to also encompass Pavlovian unconditioned stimuli.

### Data analysis

After the 5 min test, we determined the number of animals on the odor side (#_AM_), the number on the no-odor side (#_noAM_), the number of larvae on the middle stripe (#_Middle_) and the total number of larvae (#_AM_ + #_noAM_ + #_Middle_ = #_Total_). From this, we calculated the odor preference [−1; 1] as

(1) $${\text{PREF}}_{\text{COUNTED}}=\frac{\left({\#}_{\mathrm{AM}}-{\#}_{\text{no AM}}\right)}{{\#}_{\text{Total}}}.$$

During the test, we recorded larval behavior using a camera (Scout SCA1390-17FC, Basler) and custom-made software written in LabView (National Instruments). These videos were analyzed using the Multi-Worm Tracker (MWT) package, which consists of real time image-analysis (the MWT) and the offline behavioral measurement software Choreography (Swierczek et al. 2011). The data derived from Choreography was then analyzed in Matlab (MathWorks) using custom-made programs.

For each larval trajectory (Supplemental Fig. S1) Choreography outputs time-series variables that describe larval movements and postures (Supplemental Table S1). From these, we calculated additional time-series variables (Supplemental Table S2) that allowed us to determine the distance of larvae from the odor source when turning, and the orientation and bearing of larvae before and after a turn (Supplemental Tables S3):

- Turns were identified according to a method adapted from Gomez-Marin et al. (2011), relying primarily on changes in reorientation speed during turning. For a turn to be identified, reorientation speed needed to pass a set of Schmitt-trigger thresholds determined empirically (Supplemental Table S4; Ohyama et al. 2013). As larvae must bend to perform a turn, the Choreography variables of head angle, kink, and curve (Supplemental Table S1) were used on those path segments identified as turns. A turn was recorded only if these additional variables also passed a set of empirically determined thresholds (Supplemental Table S5). Only events with a change in orientation >20° were regarded as turns as preliminary analysis had indicated that events below this value displayed no bias in direction regardless of experimental conditions and thus did not contribute to the orientation behavior under study (data not shown).
- Turns were often flanked by lateral head sweeps that we call head casts. These were identified using the Choreography-variable head angle and a further set of empirical Schmitt-trigger thresholds (Supplemental Table S4). Head casts that occurred within a time window of 5 sec before a turn to 0.5 sec after a turn were classified as flanking that turn, according to Gomez-Marin et al. (2011). Notably, head casts can also take place during runs, that is, they are not necessarily flanking turns (Gomez-Marin et al. 2011; Gomez-Marin and Louis 2014).
- Runs are defined as the period between a turn and the first flanking head cast of the following turn.

From the above variables we calculated the following measurements to describe chemotaxis behavior.

- Filmed preference: the relative amount of time (*T*) larvae spent on the odor side (AM) of the Petri dish (calculated per Petri dish, *N* = 40, Fig. 1B′–D′), with odor preference [−1; 1] defined as
  (2) $${\text{PREF}}_{\text{FILMED}}=\frac{\left(T_{\mathrm{AM}}-T_{\text{no AM}}\right)}{T_{\text{Total}}}.$$
- Run speed: the average speed (mm/sec) of the larval midpoint during runs, calculated per Petri dish (*N* = 40, Fig. 2).
- Larval density: defined as the number of animals per area (mm^2^). We applied a sliding rectangular filter of 30 mm side length centered at each position (step width 2 mm).
- Turn rate: defined as the number of turns (NT) divided by the duration of time larvae were tracked (*T*):
  (3) $$\text{Turn}\;\text{rate}\;(\text{turns/min})=\frac{\text{sum}(\mathrm{NT})}{\text{sum}(T)}.$$
- The overall turn rate was calculated per Petri dish (*N* = 40). To visualize how turn rate varies with bearing to the odor source, we calculated turn rate over all bearing angles ([−180°, 180°], where 0° represents a bearing toward the odor source), with data binned every 1° and a sliding filter of ±30° applied at each step. Data were pooled from all experiments to calculate a single value (Fig. 3A–C) for each bin. As turn rate varied with the bearing angle, we determined the turn rate for bearings toward the source (absolute bearing angle <90°, Fig. 3A′–C′) and away from the source (absolute bearing angle >90°, Fig. 3A″–C″). These calculations were performed once per Petri dish (*N* = 40). To visualize how turn rate varies over both distance to the odor source and bearing we pooled all data and applied a sliding box filter of ±30° and ±15 mm at each step (step width of 2° and 2 mm).
- Proportion of turns toward odor: a turn toward the odor was defined as
  (4) Turn toward odor=absolute bearing angle after turn<absolute bearing angle before turn.
- From this, the proportion of turns toward the odor was calculated (per Petri dish, *N* = 40, Fig. 4A–C). To visualize how the proportion of turns toward the odor varies over both distance to the odor source and time, we pooled all data and applied a sliding box filter of ±22.5 sec and ±10 mm at each step (step width of 7.5 sec and 2.5 mm).
- Turning angle: we calculated the angular difference between the tail angle before and after turning. The sign of the turning angle was determined according to the bearing before the turn and the direction that the larva then implemented. When calculated over bearing angle, data were binned every 1° and a sliding filter of ±30° applied at each step. Data were pooled from all experiments to give a single value for each bin (Fig. 4A′–C′). To visualize how turning angle varies over both distance to the odor source and bearing we pooled all data and applied a sliding box filter of ±30° and ±15 mm at each step (step width of 2° and 2 mm).

### Model

For each experimental group, we separately modeled larval chemotaxis based on the empirical distribution of sensory-motor variables observed for this group, namely run speed, turn rate, and turning direction with respect to the odor gradient. The specific purpose of these deliberately minimal model simulations was to see whether modulations in these parameters indeed were sufficient to bring about the empirical between-group differences in olfactory preference.

Larvae were modeled as a jointed, two-segment object consisting of head and tail segment (Fig. 5A,B). Because the grand-average length of the larvae in our experiments was 4.3 mm, the length of each segment was set to 2.15 mm. The simulation time step (d*t*) was set to 0.0625 sec, corresponding to the sampling interval used for the experimental data. Model larvae started from positions drawn at random from the empirical start positions of the respective experimental group. Larval chemotaxis was modeled by a two-state Markov process (Norris 1997):

- Run and turn rate: while in the run state the model larvae moved in the direction of the head segment with constant run speed. Run speed was set to the median empirical run speed of the respective experimental group (see Fig. 2). At each time step a small angular noise term drawn at random (interval [−1.8°, +1.8°]) was added to the current head segment orientation, which allowed larvae to randomly reenter the arena when running along the border. Angular noise terms were redrawn whenever the head segment moved into the border of the arena, which results in the larva gradually aligning to the border as it runs into it. At each simulation time step, model animals could make a transition into the turn state with probability *P*(turn|run) = *r*_turn_ · d*t*. The probability for remaining in the run state while running equalled *P*(run|run) = 1 − *P*(turn|run). At each time step, the turn rate *r*_turn_ was set to the turn rate that was empirically observed for the respective experimental group, at the model's current bearing and distance from the odor source (as shown in Supplemental Fig. S6). The dependency of turn rate on the current bearing angle and the current distance to the odor source thus mimicked the effect of the actual sensory experience in real animals.
- Turning direction and turn actuation: whenever the model animal transitioned into the turn state, a turning angle was drawn at random from a subsample of the group's experimentally observed turning angles as follows: the preturn bearing angles at these turns were required to fall into the range [current bearing angle −30°, current bearing angle +30°], and the distance to the odor source at these turns into the range [current distance to source −15 mm, current distance to source +15 mm] (the specified ranges coincide with the filter widths in Supplemental Fig. S7 and ensure a high sample number in each subsample). This procedure of drawing a turning angle from a subsample of experimental turning angles based on bearing angle and distance to the odor source (shown in Supplemental Fig. S7) mimicked the effect of sensory input on the choice of turning direction experienced by real animals. While in the turn state the model larva rotated their head segments in the direction of the drawn turning angle with a constant angular speed of 53.7°/sec; this corresponds to the grand-average time derivative of the head angle at the onset of empirically observed events (onset was defined as the time-point where the head angle exceeded the 20° threshold). Animals remained in the turn state with probability *P*(turn|turn) = 1 as long as the absolute of the angle between the head and the tail segment was smaller than the absolute of the drawn turning angle. As soon as the designated turning angle was assumed, the state was switched to the run state with probability *P*(run|turn) = 1 and animals resumed their forward movement. In addition, the turn state was also switched to the run state with probability *P*(run|turn) = 1 whenever the head segment rotated into the border of the arena.

To estimate the relative importance of turn rate and turning direction for overall differences between experimental groups, we ran our simulations in four different modes. In the first mode (*realistic turn rate and realistic turning direction*) the simulation was run as described above, such that both the turn rate and the turning angles were drawn from the empirical data. In the second and third mode either realistic turn rate or realistic turning direction was substituted with random behavior: for the second mode (*random turn rate and realistic turning direction*) turn rate equalled the average turn rates for a given experimental condition. For the third mode (*realistic turn rate and random turning direction*) for each turn the current bearing angle was substituted by a random bearing angle (drawn from the interval [−180°, +180°]), and the current distance to the odor source was substituted with a random distance (drawn from the interval [0, 100 mm]). A turning angle then was randomly drawn from a subsample of experimental turning angles around these constraints as described above for the fully realistic model. Finally, for the fourth mode (*random turn rate and random turning direction*) both turn rate and turning direction were chosen at random as described above.

For each model mode and each experimental condition we simulated 1000 trials, where each trial consisted of a simulation of 13 animals over five minutes within a circular arena of 150 mm diameter. After the simulation, the trajectories in each trial were fragmented to match the fragmentation in the experimental data due to unresolved tracking at the boundaries of the Petri dish and during collisions: episodes of simulated trajectories were discarded at all time points at which the criteria (distance between arena center and midpoint >67 mm) was met, or the distance between the midpoints of any two model larvae was smaller than 5 mm. These settings reproduced the experimental constraints at the Petri dish boundaries, as well as the time course of the average number of tracked animals in the experiment (which was 13, as mentioned above). We did not test any of the simulated preference scores for statistical significance because at our sample size of 1000 trials the observed scores converge to their expected values.

### Statistics and graphs

Nonparametric statistics (one-sample sign test, Kruskal–Wallis test, Mann–Whitney *U*-test; OSS, KW, MWU) were applied throughout the study, using Statistica (StatSoft, Tulsa) for the PC (the one-sample sign-test uses a web-based statistic tool provided on http://www.fon.hum.uva.nl/Service/Statistics/Sign_test.html). When multiple comparisons were performed within one analysis, a Bonferroni correction was applied to keep the experiment-wide error rate below 5% by dividing the critical *P*-value by the number of tests (e.g., for three tests the adjusted *P-*value was *P* < 0.05/3). When data are displayed as box plots, the middle line shows the median, the box boundaries the 25% (*q*_1_) and 75% (*q*_3_) quantiles, and the whiskers *q*_1_ − 1.5 × (*q*_3_ − *q*_1_) and *q*_3_ + 1.5 × (*q*_3_ − *q*_1_).

## Data availability

The raw behavioral data set is available from the corresponding authors upon request.

## Supplementary Material

Supplemental Material

## References

[SCHLEYERLM037978C1] Bossing T, Udolph G, Doe CQ, Technau GM 1996 The embryonic central nervous system lineages of *Drosophila melanogaster*. I. Neuroblast lineages derived from the ventral half of the neuroectoderm. Dev Biol 179: 41–64. 8873753 10.1006/dbio.1996.0240

[SCHLEYERLM037978C2] Boyle J, Cobb M 2005 Olfactory coding in *Drosophila* larvae investigated by cross-adaptation. J Exp Biol 208: 3483–3491. 16155221 10.1242/jeb.01810

[SCHLEYERLM037978C3] Brembs B, Heisenberg M 2000 The operant and the classical in conditioned orientation of *Drosophila melanogaster* at the flight simulator. Learn Mem 7: 104–115. 10753977 10.1101/lm.7.2.104 PMC311324

[SCHLEYERLM037978C4] Chen YC, Mishra D, Schmitt L, Schmuker M, Gerber B 2011 A behavioral odor similarity “space” in larval *Drosophila*. Chem Senses 36: 237–249. 21227903 10.1093/chemse/bjq123 PMC3038273

[SCHLEYERLM037978C5] Cobb M 1999 What and how do maggots smell? Biol Rev 74: 425–459.

[SCHLEYERLM037978C6] Cobb M, Domain I 2000 Olfactory coding in a simple system: adaptation in *Drosophila* larvae. Proc Biol Sci 267: 2119–2125. 11416918 10.1098/rspb.2000.1258 PMC1690778

[SCHLEYERLM037978C7] Colomb J, Grillenzoni N, Stocker RF, Ramaekers A 2007 Complex behavioural changes after odour exposure in *Drosophila* larvae. Anim Behav 73: 587–594.

[SCHLEYERLM037978C8] Craig W 1918 Appetites and aversions as constituents of instincts. Biol Bull 34: 91–107. 10.1073/pnas.3.12.685 PMC1091358 16586767

[SCHLEYERLM037978C9] Diegelmann S, Klagges B, Michels B, Schleyer M, Gerber B 2013 Maggot learning and synapsin function. J Exp Biol 216: 939–951. 23447663 10.1242/jeb.076208

[SCHLEYERLM037978C10] Elsner B, Hommel B 2001 Effect anticipation and action control. J Exp Psychol Hum Percept Perform 27: 229–240. 11248937 10.1037//0096-1523.27.1.229

[SCHLEYERLM037978C11] Fishilevich E, Domingos AI, Asahina K, Naef F, Vosshall LB, Louis M 2005 Chemotaxis behavior mediated by single larval olfactory neurons in *Drosophila*. Curr Biol 15: 2086–2096. 16332533 10.1016/j.cub.2005.11.016

[SCHLEYERLM037978C12] Fraenkel GS, Gunn DL 1961 The orientation of animals. Dover Publications, New York.

[SCHLEYERLM037978C13] Gerber B, Hendel T 2006 Outcome expectations drive learned behaviour in larval *Drosophila*. Proc R Soc B 273: 2965–2968. 10.1098/rspb.2006.3673 PMC1639518 17015355

[SCHLEYERLM037978C14] Gerber B, Stocker RF 2007 The *Drosophila* larva as a model for studying chemosensation and chemosensory learning: a review. Chem Senses 32: 65–89. 17071942 10.1093/chemse/bjl030

[SCHLEYERLM037978C15] Gershow M, Berck M, Mathew D, Luo L, Kane EA, Carlson JR, Samuel AD 2012 Controlling airborne cues to study small animal navigation. Nat Methods 9: 290–296. 22245808 10.1038/nmeth.1853 PMC3513333

[SCHLEYERLM037978C16] Gomez-Marin A, Louis M 2012 Active sensation during orientation behavior in the *Drosophila* larva: more sense than luck. Curr Opin Neurobiol 22: 208–215. 22169055 10.1016/j.conb.2011.11.008

[SCHLEYERLM037978C17] Gomez-Marin A, Louis M 2014 Multilevel control of run orientation in *Drosophila* larval chemotaxis. Front Behav Neurosci 8: 38. 24592220 10.3389/fnbeh.2014.00038 PMC3923145

[SCHLEYERLM037978C18] Gomez-Marin A, Stephens GJ, Louis M 2011 Active sampling and decision making in *Drosophila* chemotaxis. Nat Commun 2: 441. 21863008 10.1038/ncomms1455 PMC3265367

[SCHLEYERLM037978C19] Hendel T, Michels B, Neuser K, Schipanski A, Kaun K, Sokolowski MB, Marohn F, Michel R, Heisenberg M, Gerber B 2005 The carrot, not the stick: Appetitive rather than aversive gustatory stimuli support associative olfactory learning in individually assayed *Drosophila* larvae. J Comp Physiol A Neuroethol Sens Neural Behav Physiol 191: 265–279. 15657743 10.1007/s00359-004-0574-8

[SCHLEYERLM037978C20] Hoffmann J 2003 Anticipatory behavioral control. In Anticipatory behavior in adaptive learning systems (ed. Butz MV, Sigaud O, Gerad P). Springer, Heidelberg, NY.

[SCHLEYERLM037978C21] Kane EA, Gershow M, Afonso B, Larderet I, Klein M, Carter AR, de Bivort BL, Sprecher SG, Samuel AD 2013 Sensorimotor structure of *Drosophila* larva phototaxis. Proc Natl Acad Sci 110: E3868–E3877. 24043822 10.1073/pnas.1215295110 PMC3791751

[SCHLEYERLM037978C22] Klein M, Afonso B, Vonner AJ, Hernandez-Nunez L, Berck M, Tabone CJ, Kane EA, Pieribone VA, Nitabach MN, Cardona A, 2015 Sensory determinants of behavioral dynamics in *Drosophila* thermotaxis. Proc Natl Acad Sci 112: E220–E229. 25550513 10.1073/pnas.1416212112 PMC4299240

[SCHLEYERLM037978C23] Kreher SA, Kwon JY, Carlson JR 2005 The molecular basis of odor coding in the *Drosophila* larva. Neuron 46: 445–456. 15882644 10.1016/j.neuron.2005.04.007

[SCHLEYERLM037978C24] Kreher SA, Mathew D, Kim J, Carlson JR 2008 Translation of sensory input into behavioral output via an olfactory system. Neuron 59: 110–124. 18614033 10.1016/j.neuron.2008.06.010 PMC2496968

[SCHLEYERLM037978C25] Lahiri S, Shen K, Klein M, Tang A, Kane E, Gershow M, Garrity P, Samuel AD 2011 Two alternating motor programs drive navigation in *Drosophila* larva. PLoS One 6: e23180. 21858019 10.1371/journal.pone.0023180 PMC3156121

[SCHLEYERLM037978C26] Larkin A, Karak S, Priya R, Das A, Ayyub C, Ito K, Rodrigues V, Ramaswami M 2010 Central synaptic mechanisms underlie short-term olfactory habituation in *Drosophila* larvae. Learn Mem 17: 645–653. 21106688 10.1101/lm.1839010

[SCHLEYERLM037978C27] Larsen C, Shy D, Spindler SR, Fung S, Pereanu W, Younossi-Hartenstein A, Hartenstein V 2009 Patterns of growth, axonal extension and axonal arborization of neuronal lineages in the developing *Drosophila* brain. Dev Biol 335: 289–304. 19538956 10.1016/j.ydbio.2009.06.015 PMC2785225

[SCHLEYERLM037978C28] Lieberman DA 2004 Learning and memory: an integrative apporach. Wadsworth Thomson, Belmont, CA.

[SCHLEYERLM037978C29] Louis M, Piccinotti S, Vosshall LB 2008 High-resolution measurement of odor-driven behavior in *Drosophila* larvae. J Vis Exp. 10.3791/638 PMC2582838 19066557

[SCHLEYERLM037978C30] Luo L, Gershow M, Rosenzweig M, Kang K, Fang-Yen C, Garrity PA, Samuel AD 2010 Navigational decision making in *Drosophila* thermotaxis. J Neurosci 30: 4261–4272. 20335462 10.1523/JNEUROSCI.4090-09.2010 PMC2871401

[SCHLEYERLM037978C31] Mishra D, Chen YC, Yarali A, Oguz T, Gerber B 2013 Olfactory memories are intensity specific in larval *Drosophila*. J Exp Biol 216: 1552–1560. 23596280 10.1242/jeb.082222

[SCHLEYERLM037978C32] Neuser K, Husse J, Stock P, Gerber B 2005 Appetitive olfactory learning in *Drosophila* larvae: effects of repetition, reward strength, age, gender, assay type and memory span. Anim Behav 69: 891–898.

[SCHLEYERLM037978C33] Norris JR 1997 Markov chains. Cambridge University Press, Cambridge, UK.

[SCHLEYERLM037978C34] Ohyama T, Jovanic T, Denisov G, Dang TC, Hoffmann D, Kerr RA, Zlatic M 2013 High-throughput analysis of stimulus-evoked behaviors in *Drosophila* larva reveals multiple modality-specific escape strategies. PLoS One 8: e71706. 23977118 10.1371/journal.pone.0071706 PMC3748116

[SCHLEYERLM037978C35] Quinn WG, Harris WA, Benzer S 1974 Conditioned behavior in *Drosophila melanogaster*. Proc Natl Acad Sci 71: 708–712. 4207071 10.1073/pnas.71.3.708 PMC388082

[SCHLEYERLM037978C36] Rescorla RA 1967 Pavlovian conditioning and its proper control procedures. Psychol Rev 74: 71–80. 5341445 10.1037/h0024109

[SCHLEYERLM037978C37] Saumweber T, Husse J, Gerber B 2011a Innate attractiveness and associative learnability of odors can be dissociated in larval *Drosophila*. Chem Senses 36: 223–235. 21227902 10.1093/chemse/bjq128 PMC3038274

[SCHLEYERLM037978C45] Saumweber T, Weyhersmüller A, Hallermann S, Diegelmann S, Michels B, Bucher D, Funk N, Reisch D, Krohne G, Wegener S, Buchner E, Gerber B 2011b Behavioral and synaptic plasticity are impaired upon lack of the synaptic protein SAP47. J Neurosci 31: 350–3518. 10.1523/JNEUROSCI.2646-10.2011 PMC6623913 21368063

[SCHLEYERLM037978C38] Scherer S, Stocker RF, Gerber B 2003 Olfactory learning in individually assayed *Drosophila* larvae. Learn Mem 10: 217–225. 12773586 10.1101/lm.57903 PMC202312

[SCHLEYERLM037978C39] Schleyer M 2009 “Formation and expression of olfactory memory in fruit fly larvae: a behaviour-based model.” Diploma thesis, Universität Würzburg.

[SCHLEYERLM037978C40] Schleyer M, Saumweber T, Nahrendorf W, Fischer B, von Alpen D, Pauls D, Thum A, Gerber B 2011 A behavior-based circuit model of how outcome expectations organize learned behavior in larval *Drosophila*. Learn Mem 18: 639–653. 21946956 10.1101/lm.2163411

[SCHLEYERLM037978C41] Schleyer M, Diegelmann S, Michels B, Saumweber T, Gerber B 2013 ‘Decision-making’ in larval *Drosophila*. In Invertebrate learning and memory (ed. Menzel R, Benjamin P), pp. 41–55 Elsevier, München.

[SCHLEYERLM037978C42] Schleyer M, Miura D, Tanimura T, Gerber B 2015 Learning the specific quality of taste reinforcement in larval *Drosophila*. Elife 4: e04711. 10.7554/eLife.04711 PMC4302267 25622533

[SCHLEYERLM037978C43] Swierczek NA, Giles AC, Rankin CH, Kerr RA 2011 High-throughput behavioral analysis in *C. elegans*. Nat Methods 8: 592–598. 21642964 10.1038/nmeth.1625 PMC3128206

[SCHLEYERLM037978C44] Twick I, Lee JA, Ramaswami M 2014 Olfactory habituation in *Drosophila*-odor encoding and its plasticity in the antennal lobe. Prog Brain Res 208: 3–38. 24767477 10.1016/B978-0-444-63350-7.00001-2

