# Supplementary material for: The impact of odor–reward memory on chemotaxis in larval Drosophila
Source: Learn Mem. 2015 May;22(5):267–77. doi: 10.1101/lm.037978.114 (PMC4408773; doi:10.1101/lm.037978.114)
Supplement: Supplemental Material [file supp_22_5_267__index.html]

Supplemental Material 

# The impact of odor–reward memory on chemotaxis in larval *Drosophila*

## Supplemental Material

**Files in this Data Supplement:**

- Supp Fig1.pdf
- Supp Fig5 - overall turn rate.pdf
- Supp Fig7 - turn direction heatmap.pdf
- Supp Fig8 - density and turn high heatmap jet.pdf
- Supp Fig6.pdf
- Supp Fig2-fru comparisons.pdf
- Supp Fig3 - speed heatmap.pdf
- Supp Fig4 - when to turn boxplot sidebyside.pdf
- Supp Movie 2.mp4
- Supp Legends.docx
- Supp Movie 1.mp4
- Supp Tables S1-S5.docx
- Supp Movie 3.mp4
