# Supplementary figures and images for: The impact of odor–reward memory on chemotaxis in larval Drosophila
Source: Learn Mem. 2015 May;22(5):267–77. doi: 10.1101/lm.037978.114 (PMC4408773; doi:10.1101/lm.037978.114)

(A)

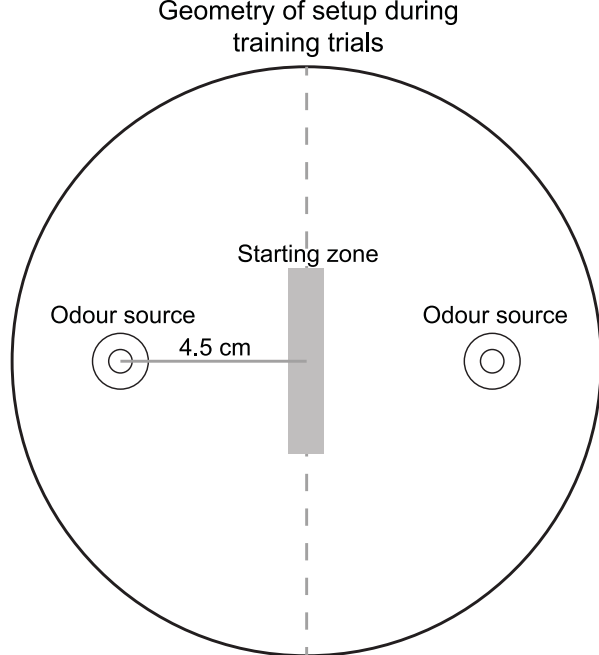

Geometry of setup during testing trials

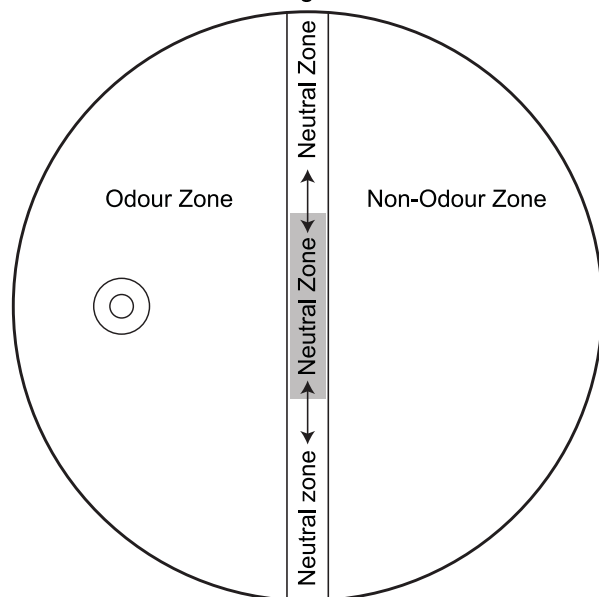

(B)

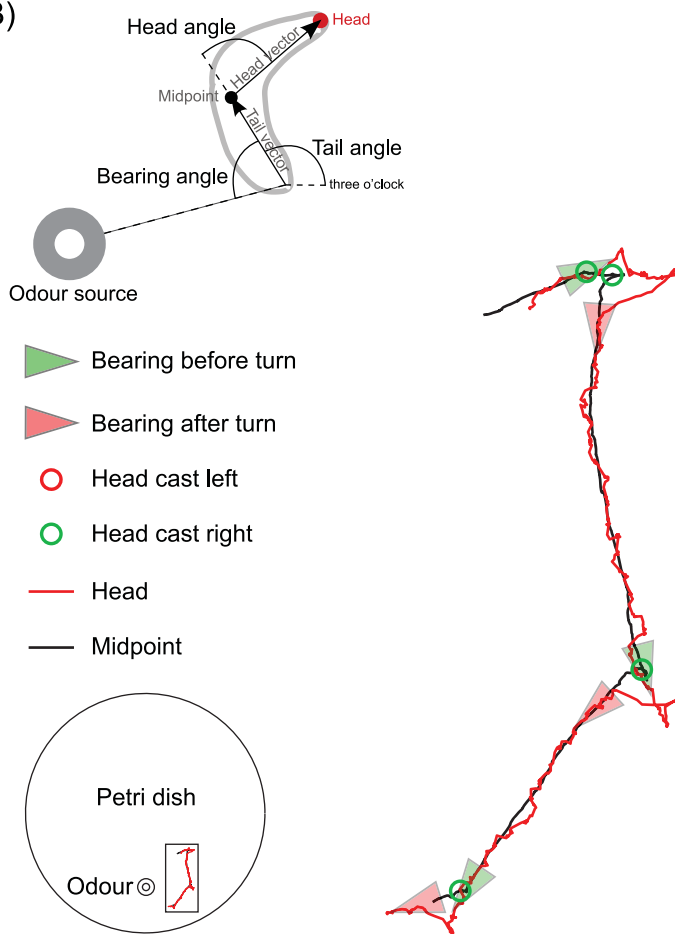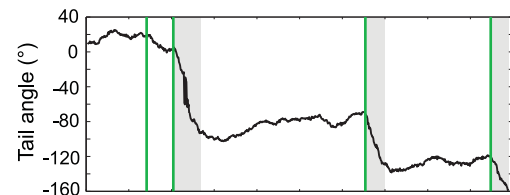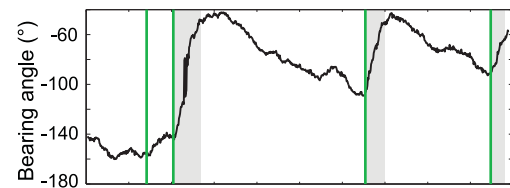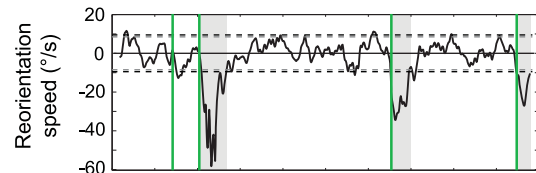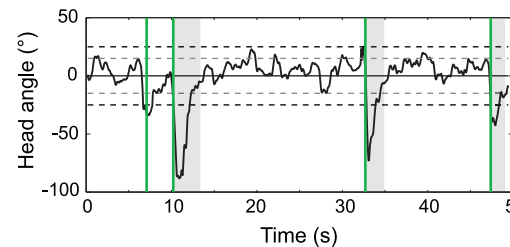

Supplement: Supplemental Material [file supp_22.5.267_Supp_Fig1.pdf]

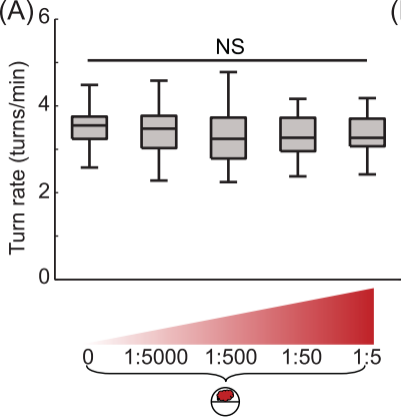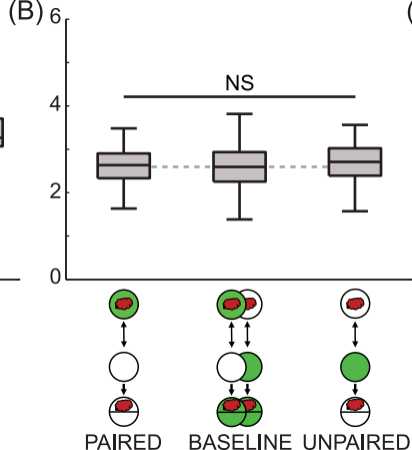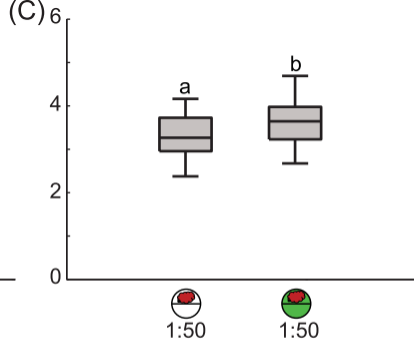

Supplement: Supplemental Material [file supp_22.5.267_SuppFig5_-_overall_turn_rate.pdf]

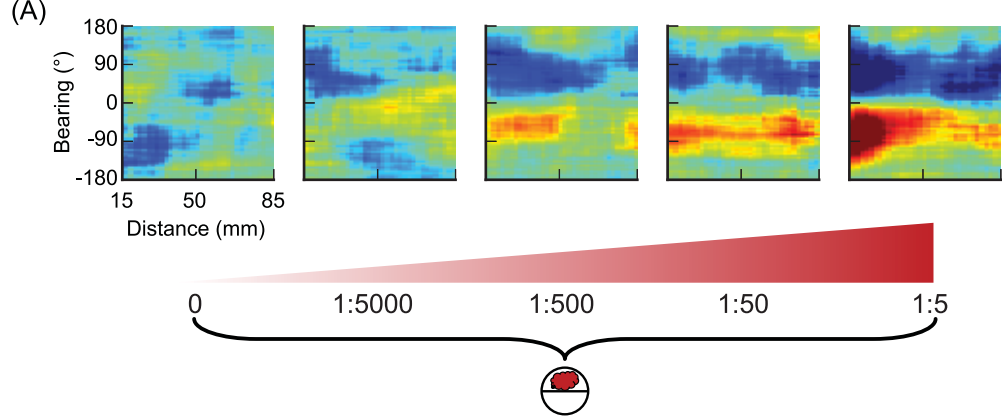

(B)

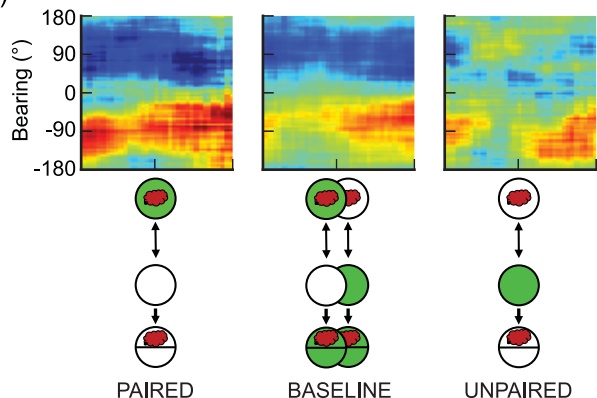

(C)

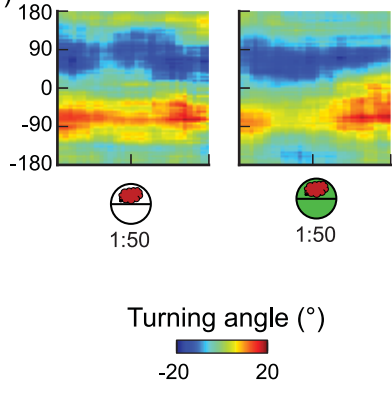

Supplement: Supplemental Material [file supp_22.5.267_SuppFig7_-_turn_direction_heatmap.pdf]

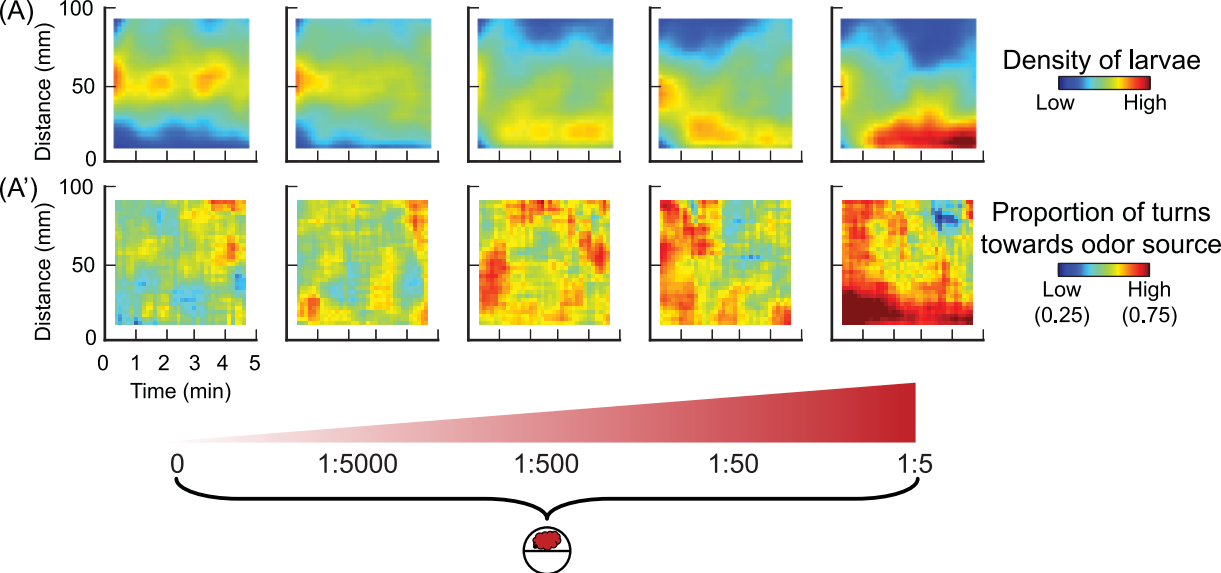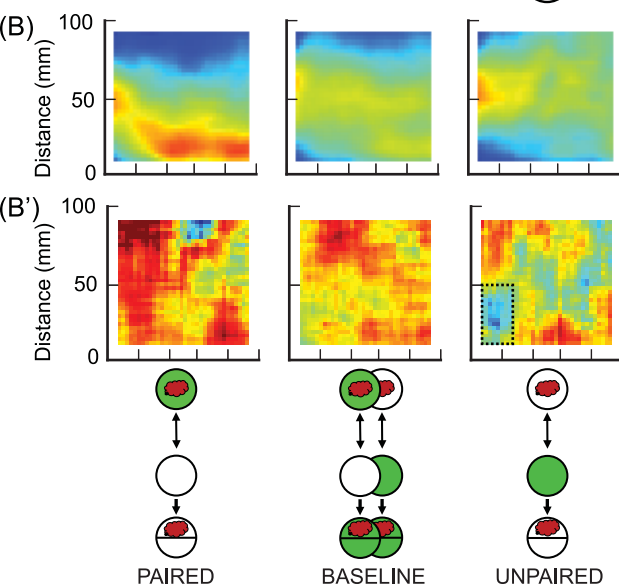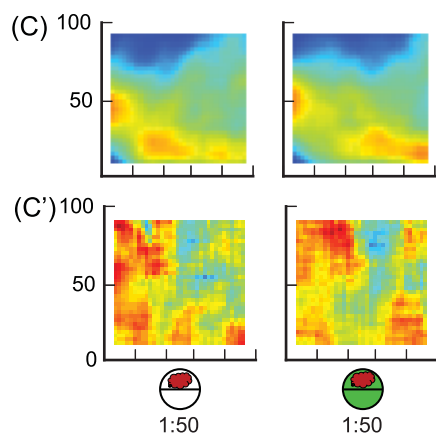

Supplement: Supplemental Material [file supp_22.5.267_SuppFig8_-_density_and_turn_high_heatmap__jet.pdf]

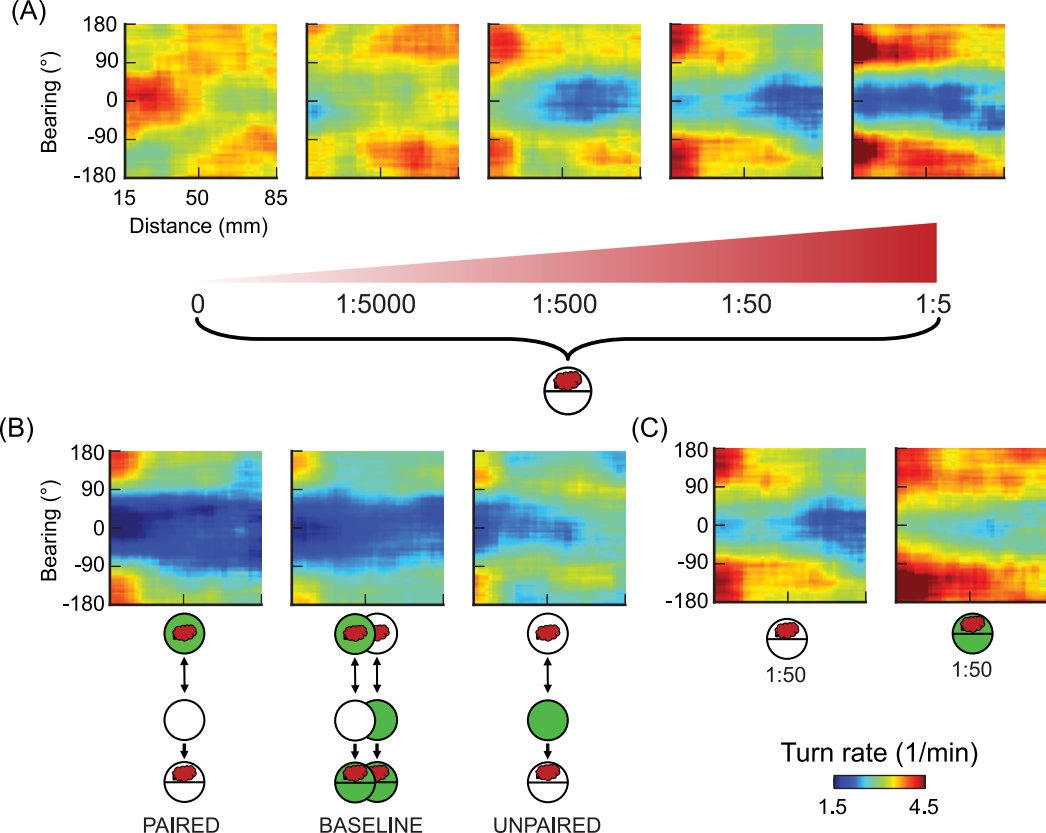

Supplement: Supplemental Material [file supp_22.5.267_Supp_Fig6.pdf]

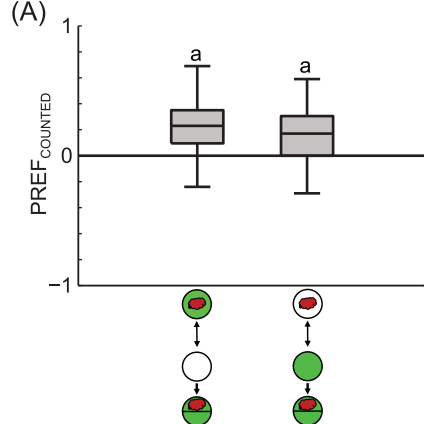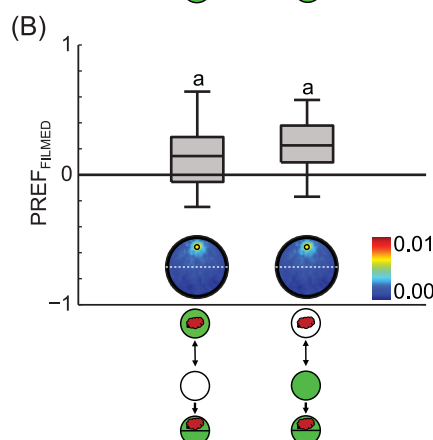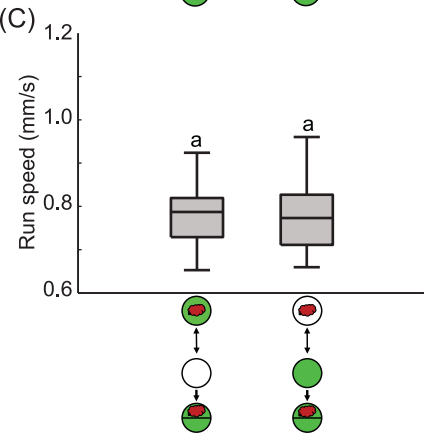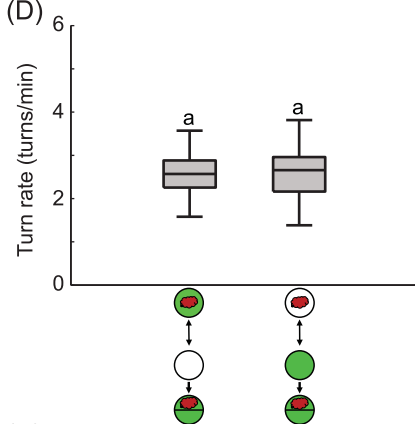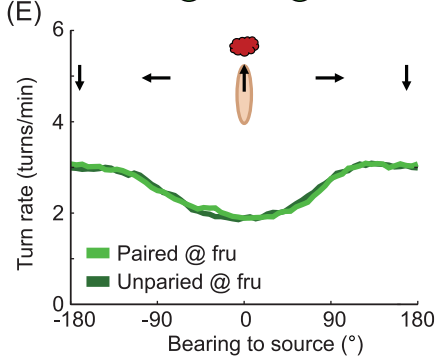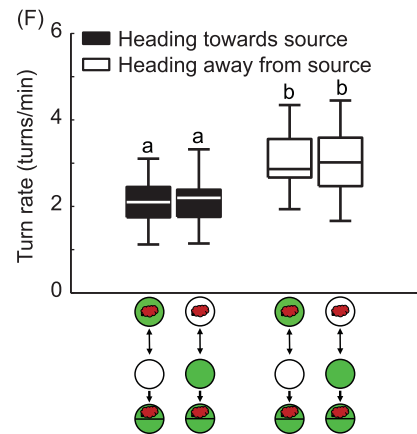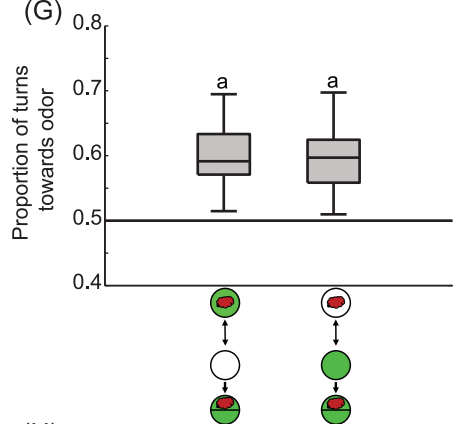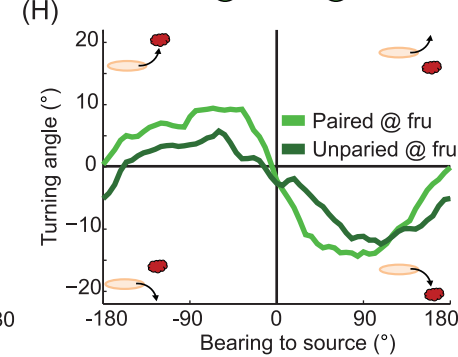

Supplement: Supplemental Material [file supp_22.5.267_Supp_Fig2_-_fru_comparisons.pdf]

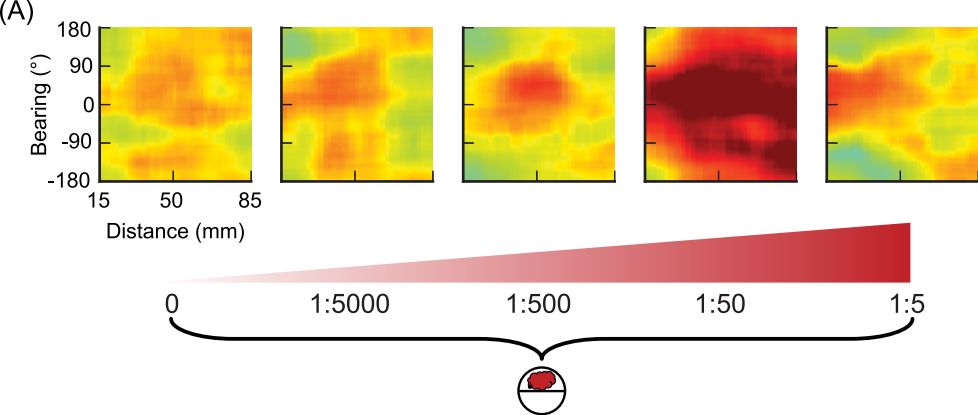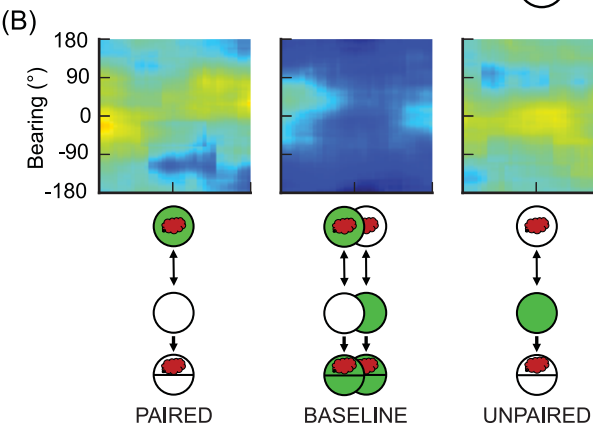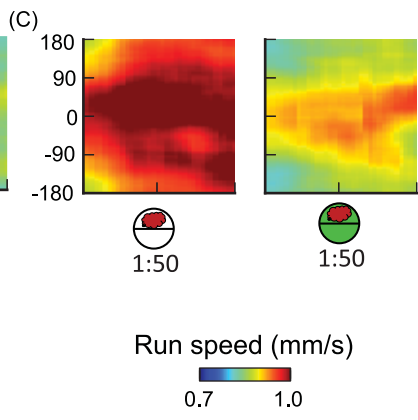

Supplement: Supplemental Material [file supp_22.5.267_SuppFig3_-_speed_heatmap.pdf]

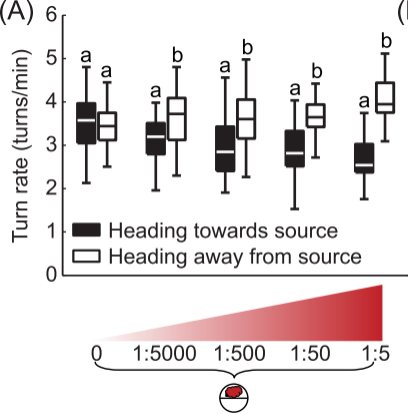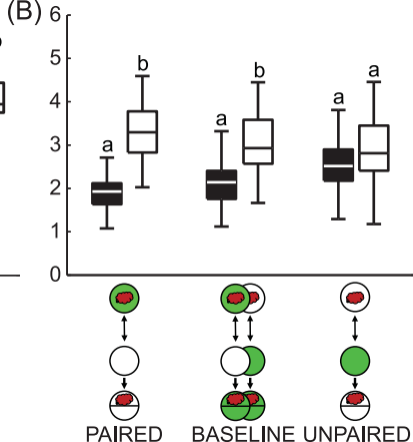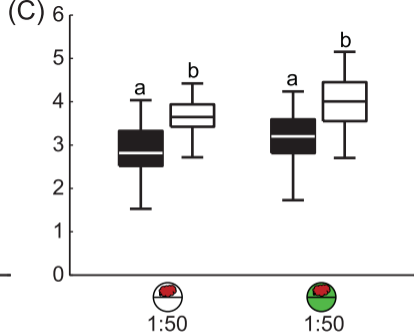

Supplement: Supplemental Material [file supp_22.5.267_SuppFig4_-_when_to_turn_boxplot_sidebyside.pdf]
