# Supplementary material for: The impact of odor–reward memory on chemotaxis in larval Drosophila
Source: Learn Mem. 2015 May;22(5):267–77. doi: 10.1101/lm.037978.114 (PMC4408773; doi:10.1101/lm.037978.114)
Supplement: Supplemental Material [file supp_22.5.267_SuppLegends.docx]

**Supplementary Material**

**Supplementary Tables 1-5**

**Table S1:** Primary time-series variables as computed by Choreography for each trajectory.

**Table S2:** Secondary time-series variables computed for each trajectory.

**Table S3:** Variables computed for each turn. Turns were identified automatically according to Gomez-Marin et al. (2011). See methods and Table S4 for more detail.

**Table S4:** Schmitt trigger thresholds for turns and casts. A turn or cast was identified when the reorientation speed (turns) or head angle (casts) crossed the high threshold value, remained above the low threshold value for a specified time (width), and did not occur within a given time/distance window of a similar event immediately preceding it (gap).

**Table S5:** Additional thresholds used to refine turn identification. A turn was only recorded if the Choreography variables of head angle, kink and curve passed this additional set of empirically determined thresholds.

**Supplementary Movies 1-3**

Time-resolved larval density on the petri dish for innate (Innate_odour_concentrations.mp4, Innate_pure_fructose.mp4) and trained groups (Paired_Baseline_Unpaired.mp4). Each movie frame shows a heatmap of larval density for a 10 s time interval (indicated by the blue bar moving along the time axis). Each frame was filtered with a 20 mm box filter and normalized such that the summation over all points equalled one.

**Figure S1: Schematic of assay geometry and data analyses**

**(A)** Assay geometry. The training and testing of larvae was carried out in 15 cm diameter Petri dishes, filled with either 1 % agarose or with 0.2 M fructose added to the agarose (according to the experimental group, see methods for more details). The odour (*n*-amyl acetate) was delivered by placing a 10 µl droplet within a reinforcement ring fixed onto the inner side of the Petri dish lid. During training two odour sources were used to ensure odour was present throughout the arena (left schematic). A group of ~20 larvae was placed in a 4.50 x 0.85 cm starting zone of the Petri dish and underwent training as described in the methods. During the test, only one odour source was used in order to create a choice situation (right schematic). Larval behaviour was filmed for 5 min from below for offline analyses (see (B) below). After 5 min the dish was removed and the position of larvae was scored as being either on the odour side of the dish, the no-odour side, or in a 1 cm-wide neutral zone.

**(B)** Data analysis. The schematic of the larva and odour source show the geometric relationship between variables. A short example trajectory is shown, highlighting the most important features identified by the analysis. The black trace shows the position of the midpoint throughout the trajectory, and the red trace that of the head. Green and red circles show the position of the midpoint at head cast onset. Green and red triangles depict the tail angle before and after a turn, respectively (see also Table S3). The four subplots on the right show some of the time-series variables for the example trajectory. The horizontal lines in the reorientation speed and head angle subplots depict the Schmitt-trigger thresholds used to identify turns and casts, respectively (see also Table S4). Green vertical lines represent head cast onset and grey shading turn duration (from turn onset to offset).

**Figure S2: Comparison of reciprocally trained groups tested on fructose**

In presence of fructose, larvae behave the same after paired and unpaired training, in terms of **(A)** endpoint-counting preference, **(B)** filmed preference, **(C)** run speed, **(D-F)** turn rate, and **(G-H)** turning direction (*P >* 0.05/7, [A] U = 585, [B] U = 566, [C] U = 800, [D] U = 788, [F] U = 792, 792, [G] U = 750, MWU). Therefore, both groups are pooled and called ‘Baseline’ in the main figures: (A) corresponds to Fig. 1C, (B) to Fig. 1C’, (C) to Fig. 2B, (D) to Fig. S5B, (E) to Fig. 3B, (F) to Fig. 3B’-B’’, (G) to Fig. 4B, and (H) to Fig. 4B’.

**Figure S3: Run speed separated by bearing angle and distance to the odour source**

Colour coded run speed as a function of bearing and distance to the odour source, separately for **(A)** different odour concentrations, **(B)** after training and **(C)** in presence or absence of sugar. There appears to be little effect of either bearing direction or distance to the source on run speed. However, run speed does vary across experimental groups, in particular in relation to the presence or absence of sugar (c.f. Fig. 2). For each condition, we pooled all data and calculated the run speed over all bearing angles ([-180°, 180°], where 0° represents a bearing towards the odour source), applying a sliding box filter of ± 30° and ± 15 mm at each step (step width of 2° and 2 mm).

**Figure S4: Turn rate when heading towards *versus* heading away from odour**

Turn rate of animals heading towards *versus* heading away from the odour source.

**(A)** The difference in turn rate when heading towards *versus* away from the odour source apparently increases with increasing odour concentration, and is significant for all odour concentrations (*P <* 0.05/5, from left to right: U = 444, 313, 277, 29, MWU), but not for the no odour condition (*P >* 0.05/5, U = 717, MWU).

**(B)** After training, turn rate differs significantly between animals heading towards *versus* away from odour in the case of both paired training (*P <* 0.05/3, U = 77, MWU) and baseline (*P <* 0.05/3, U = 904, MWU), but not after unpaired training (*P >* 0.05/3, U = 578, MWU).

**(C)** Both in presence and absence of fructose turn rate is increased when animals heading away from the odour source as compared to animals heading towards the odour (*P <* 0.05/2, from left to right: U = 277, 260, MWU).

Significant differences between heading towards the source and heading away from the source for each condition (Mann-Whitney U-tests) are indicated with lower case letters above the boxes. For other details, see legend of Fig. 1.

**Figure S5: Total turn rate**

**(A)** Average turn rate is neither influenced by the concentration of *n*-amylacetate (*P >* 0.05, H = 4.9, df = 4, KW) nor **(B)** by training experience (*P >* 0.05, H = 2.3, df = 2, KW), but **(C)** is increased by the presence of fructose (*P <* 0.05, U = 524, MWU).

Significant differences (Mann-Whitney U-tests) are indicated with lower case letters above the boxes. For other details, see legend of Fig. 1.

**Figure S6: Turn rate separated by bearing angle and distance to the odour source**

Colour coded turn rate as a function of bearing and distance to the odour source, separately for **(A)** different odour concentrations, **(B)** after training and **(C)** in presence or absence of sugar. Turn rate varies with both bearing direction and distance to the odour source, as well as between experimental groups (c.f. Fig. 3). For each condition, we pooled all data and calculated the turn rate over all bearing angles ([-180°, 180°], where 0° represents a bearing towards the odour source), applying a sliding box filter of ± 30° and ± 15 mm at each step (step width of 2° and 2 mm).

For simulating the model, the turn rate was set to that turn rate that was empirically observed for the respective experimental group above, at the model’s current bearing and distance from the odour source. See methods for more details.

**Figure S7: Turning angle separated by bearing angle and distance to the odour source**

Colour coded turning angle as a function of bearing and distance to the odour source, separately for **(A)** different odour concentrations, **(B)** after training and **(C)** in presence or absence of sugar. Turning angle varies with bearing direction but not appear to vary with distance to the odour source. There is a clear difference between conditions (c.f. Fig. 4). For each condition, we pooled all data and calculated the turning angle over all bearing angles ([-180°, 180°], where 0° represents a bearing towards the odour source), applying a sliding box filter of ± 30° and ± 15 mm at each step (step width of 2° and 2 mm).

For simulating the model, turning angle was drawn at random from a subsample of experimentally observed turning angles in the respective group. The subsample of turning angles was chosen as follows: the pre-turn bearing angles at these turns were required to fall into the range [current bearing angle – 30°, current bearing angle + 30°], and the distance to the odour source at these turns into the range [current distance to source –15 mm, current distance to source + 15 mm]. See methods for more details.

**Figure S8: Evidence suggesting odour repulsion after unpaired training**

Colour coded density of larvae **(A-C)** and proportion of turns towards the odour source **(A’-C’)** across experimental conditions. Data are plotted as function of time (x-axis) and distance from odour source (Y-axis).

The proportion of turns towards the odour source is increased by increasing odour concentration (A’). Relative to baseline, the same is seen after paired training (B’). In both cases, this leads to a quick accumulation at the odour source (A, B).

Interestingly, within the first one and a half minutes after unpaired training, larvae bias their turns *away* from the odour source when close to it (stippled box in B’, right-most panel). This is not seen for any other experimental condition. Such turning away could be the basis for odour repulsion upon unpaired training as described in terms of negative PREF scores in e.g. Chen et al. (2011), Saumweber et al. (2011), Schleyer et al. (2011), Mishra et al. (2013).
