# Supplementary material for: The impact of odor–reward memory on chemotaxis in larval Drosophila
Source: Learn Mem. 2015 May;22(5):267–77. doi: 10.1101/lm.037978.114 (PMC4408773; doi:10.1101/lm.037978.114)
Supplement: Supplemental Material [file supp_22.5.267_TablesS1_S5.docx]

**Table S1**

| Variable name | Units | Description |
| --- | --- | --- |
| Time | *s* | *Time of sample point* |
| Midpoint x | *mm* | *x coordinate of the midpoint* |
| Midpoint y | *mm* | *y coordinate of the midpoint* |
| Head x | *mm* | *x coordinate of the head* |
| Head y | *mm* | *y coordinate of the head* |
| Head vector x | *mm* | *x-component of the head vector* |
| Head vector y | *mm* | *y-component of the head vector* |
| Tail vector x | *mm* | *x-component of the tail vector* |
| Tail vector x | *mm* | *y-component of the tail vector* |
| Head angle | *°* | *The angle formed between the head vector and the tail vector* |
| Kink | *rad* | *The angle between the line from the first to third point of the skeleton and the four through last points* |
| Curve | *rad* | *The average angle of points along the object’s skeleton* |

**Table S2**

| Variable name | Units | Description |
| --- | --- | --- |
| Speed | *mm/s* | *Speed of the midpoint* |
| Path length | *mm* | *Path length travelled by the animal* |
| Distance to the odour | *mm* | *Distance from the midpoint to the odour source* |
| Tail angle | *°* | *Angle between the tail vector and 3 o’clock* |
| Reorientation speed | *°/s* | *The derivative of the tail angle* |
| Bearing angle | *°* | *Angle between the tail vector and the vector pointing from the midpoint to the odour source* |

**Table S3**

| Variable name | Units | Description |
| --- | --- | --- |
| Distance to odour at turn | *mm* | *Distance to the odour at turn onset* |
| Tail angle before a turn | *°* | *Tail angle 1.5s – 2.5s before a turn. Tail angle value taken at minimum head angle during this window.* |
| Tail angle after a turn | *°* | *Tail angle 1.5s – 2.5s after a turn. Tail angle value taken at minimum head angle during this window.* |
| Bearing before a turn | *°* | *Bearing angle 1.5s – 2.5s before a turn. Bearing angle value taken at minimum head angle during this window.* |
| Bearing after a turn | *°* | *Bearing angle 1.5s – 2.5s after a turn. Bearing angle value taken at minimum head angle during this window.* |

**Table S4**

| Behaviour | High threshold | Low threshold | Width | Gap |
| --- | --- | --- | --- | --- |
| Turn | 9.5 °/s | 8.5 °/s | 0.8s | 0.8s *or* 1mm |
| Cast | 25.0 ° | 15.0 ° | 0.3s | 0.2s |

**Table S5**

| Behaviour | Threshold | Width |
| --- | --- | --- |
| Head angle | 20 ° | 0.3125s |
| Curve | 1.1 rad | 0.25s |
| Kink | 1.1 rad | 0.25s |
